# Plasma proteomic signatures of preclinical Alzheimer’s disease in clinically unimpaired older adults

**DOI:** 10.1186/s13024-026-00941-5

**Published:** 2026-04-24

**Authors:** Alexandra N. Trelle, Karly A. Cody, Tran T. Nguyen, Joseph R. Winer, Skylar Weiss, Isha Sai, Tyler Ward, Gloria Cheng, Divya Channappa, Justin Mendiola, Amal Al-Rajhi, Keerthana Raghuraman, Sharon J. Sha, Edward N. Wilson, Tony Wyss-Coray, Holden T. Maecker, Anthony D. Wagner, Elizabeth C. Mormino

**Affiliations:** 1 Department of Neurology & Neurological Sciences, Stanford University School of Medicine, Stanford, CA 94305 USA; 2 Institute for Immunity, Transplantation, and Infection Operations, Stanford University School of Medicine, Stanford, CA 94305 USA; 3 Department of Psychology, Stanford University, Stanford, CA 94305 USA; 4 Wu Tsai Neurosciences Institute, Stanford University, Stanford, CA 94305 USA; 5 Phil and Penny Knight Initiative for Brain Resilience, Stanford University, Stanford, CA 94305 USA; 6 Department of Microbiology and Immunology, Stanford University School of Medicine, Stanford, CA 94305 USA; 7 Molecular Imaging Program at Stanford (MIPS), Stanford University, Stanford, CA 94305 USA

**Keywords:** Preclinical Alzheimer’s disease, Plasma biomarkers, Proteomics, Brain-derived pTau, Amyloid pathology, Tau pathology, Inflammation, NUcleic acid-linked immuno-sandwich assay (NULISA), NULISA with next-generation sequencing readout (NULISAseq), Lumipulse

## Abstract

**Background:**

Multi-analyte plasma proteomic panels that can accurately detect initial Alzheimer’s disease (AD) pathology in preclinical populations and simultaneously measure related biological processes relevant for disease risk are critical for advancing early detection and prognosis.

**Methods:**

Using the NULISAseq™ CNS panel, we measured plasma proteomics from 315 clinically unimpaired (CU) older adults across two independent cohorts: the Stanford Aging and Memory Study (SAMS; *n* = 193) with paired cerebrospinal fluid (CSF) and plasma analyzed with Lumipulse, and the Attention, Memory, and Aging Study at Stanford (AMASS; *n* = 122) with paired florbetaben (FBB) amyloid PET. We evaluated correspondence of core AD-relevant biomarkers pTau217, pTau181, Aβ42/Aβ40, pTau217/Aβ42, GFAP, and NfL measured using multiplex NULISAseq and single-plex Lumipulse immunoassays. ROC curve analyses compared performance for detecting amyloid-positivity (A+) (a) across platforms in SAMS and (b) across brain-derived (BD) and total-pTau assays in AMASS, leveraging novel NULISAseq immunoassays. Linear models were applied across all NULISAseq CNS proteins to explore proteomic abundance patterns associated with age, sex, *APOE-*ε4, amyloid burden (CSF Aβ42/Aβ40, amyloid PET), and tau burden (CSF pTau181, PI-2620 tau PET) using an FDR-corrected *p*-value of < 0.05 to identify significant targets.

**Results:**

In SAMS, moderate to high correlations were observed between NULISAseq and Lumipulse plasma biomarkers. NULISAseq pTau217/Aβ42 (AUC: 0.940) and pTau217 (AUC: 0.879) performed as well as corresponding single-plex Lumipulse assays (pTau217/Aβ42, AUC: 0.907; pTau217, AUC: 0.838) for detecting CSF A+ in SAMS. In AMASS, BD-pTau217 (AUC: 0.920) and BD-pTau181 (AUC: 0.920) exhibited the highest performance in discriminating PET A+, providing significant performance gains compared to total-pTau measures (pTau217, AUC: 0.861; pTau181, AUC: 0.763). Exploratory proteomic abundance analyses across NULISA CNS targets revealed pTau isoforms as most differentially expressed with amyloid burden across cohorts, together with upregulation of GFAP and downregulation of Aβ42 in SAMS. Tau burden was associated with upregulation of plasma pTau217, independent of amyloid burden, together with proteins related to astrocyte activation, inflammation, and synaptic integrity.

**Conclusions:**

NULISAseq multiplex immunoassays, including novel BD-pTau assays, accurately detect AD pathology among CU older adults and identify multiple biological pathways related to aging and early biomarker abnormality that may become dysregulated in preclinical AD.

**Supplementary Information:**

The online version contains supplementary material available at 10.1186/s13024-026-00941-5.

## Introduction

Effective diagnosis, treatment, and prevention of Alzheimer’s disease (AD), particularly in the era of disease-modifying therapeutics, relies critically on the availability of accessible and scalable biomarkers that can accurately detect AD pathology in vivo. The recent development of blood-based biomarkers that can accurately detect cerebral amyloid pathology is transforming the landscape of AD research and clinical trial enrollment by reducing key barriers to large-scale screening for biomarker-determined eligibility [1, 2]. High performing plasma assays of pTau217 and Aβ42/Aβ40 that perform comparably to gold standard CSF Aβ42/Aβ40 and amyloid PET imaging for detecting abnormal amyloid are now commercially available [3], including those based on mass spectrometry [2] (e.g., C_2_N Diagnostics) as well as immunoassays such as those from Fujirebio Diagnostics on the fully-automated Lumipulse platform [4]. Blood-based biomarkers are also on a trajectory to transform clinical care by supporting more accurate diagnosis and determining eligibility for therapeautics [5, 6]. In a landmark decision, the Lumipulse pTau217/Aβ42 ratio from Fujirebio was the first blood-based biomarker to be cleared by the FDA to support AD diagnosis in clinically impaired individuals, setting the stage for future approval of other high performing plasma assays. Given that amyloid positive (A+) clinically unimpaired (CU) individuals are at increased risk for cognitive decline [7] and interventions may be most successful when implemented early, it is critical that blood-based biomarkers are sensitive to early and subtle AD pathology in older CU populations.

Critically, amyloid and tau biomarkers offer only a partial picture of the complex underlying pathophysiology of AD and risk of clinical progression. For example, the presence of co-pathology such as vascular injury, ⍺-synuclein, and TDP-43 is common and impacts clinical trajectories [8]. Moreover, mounting evidence points to the relevance of additional biological processes that are not specific to AD, with markers of neuronal injury (e.g., neurofilament light chain, NfL), neuroinflammation (e.g., glial fibrillary acidic protein, GFAP), and synaptic integrity (synaptosomal-associated protein 25, SNAP-25) providing unique information regarding disease stage and risk for clinical progression [9–12]. Given the relevance of a growing number of plasma biomarkers, the need for multi-analyte plasma biomarker panels that can measure multiple proteins simultaneously (i.e., multiplexing) is increasingly evident. For example, targeted multi-analyte panels could enable enhanced biomarker staging and theragnostic evaluation of possible interventions, both in clinical settings for treatment as well as in research settings to facilitate trial enrollment, advancing the development of personalized precision medicine.

With the growing promise of scalable plasma biomarkers in research and clinical practice, addressing specificity for AD-related pathology and potential measurement confounds remains critical. Indeed, recent evidence indicates that plasma pTau species can originate both from the CNS and peripheral sources [13] and exhibit elevations in patients with amyotrophic lateral sclerosis (ALS) linked to peripheral nerve damage [14]. Critically, the protein structure of pTau originating from peripheral sources, also known as high-molecular-weight (HMW) isoforms, contains a large peptide insert (exon 4a) that is absent from pTau originating from the CNS (i.e., brain-derived (BD) pTau), which predominantly expresses low-molecular-weight (LMW) isoforms [15]. While most available immunoassays recognize N-terminal epitopes present in both forms of pTau and therefore lack specificity regarding protein origin, existing evidence from assays that target regions present only in LMW tau isoforms expressed in the CNS indicates they provide better discrimination of AD versus ALS [16]. Due to reduced interference from peripheral sources, assays that target BD-pTau isoforms may offer enhanced sensitivity and specificity for AD pathology and improve real-world performance and utility.

The recently developed NULISA (Nucleic acid-Linked Immuno-Sandwich Assay) [17] multiplex plasma proteomics platform can simultaneously measure over 120 CNS targets, including core Aβ and pTau isoforms together with proteins related to inflammation, synaptic integrity, and neuronal injury. Initial studies benchmarking NULISA’s CNS panel suggest that it performs well in measuring core AD-relevant biomarkers (pTau217, pTau231, pTau181, Aβ42, NfL, GFAP) relative to single-plex immunoassays [18–20] and exhibits strong correspondence with high throughput methods such as SomaScan for established markers of neuronal injury (NfL, SNAP-25, NRGN) and inflammation (GFAP, TREM2, CHI3L1) [18, 20]. Moreover, NULISAseq plasma pTau217 performs comparably with Simoa plasma pTau217 for detection of amyloid PET positivity [19, 21, 22]. These early findings suggest that the NULISA multiplex platform may be well-suited to support quantification of established AD biomarkers together with measurement of related processes that are relevant for disease progression and monitoring. Critically, the recently updated NULISA CNS panel now also includes measures of BD-pTau217, BD-pTau181, and BD-pTau231, leveraging a novel assay that utilizes detection antibodies that target the LMW-specific region of the protein structure, enabling critical comparisons between total-pTau and BD-pTau isoforms measured within the same panel. With these rapid advancements in plasma multiplexing, including novel BD-pTau assays, it remains critical to evaluate these tools through the lens of early detection in CU aging cohorts.

In this study, we leveraged two independent biomarker-characterized CU cohorts to further evaluate the performance of the NULISAseq CNS panel for early detection of AD pathology and to explore proteomic abundance patterns associated with AD-related phenotypes among CU. In the Stanford Aging and Memory Study (SAMS) cohort with paired plasma measured using single-plex immunoassays on the Lumipulse platform [4, 23], we evaluated correspondence between measurements of core AD-relevant biomarkers pTau217, pTau181, Aβ42/Aβ40, pTau217/Aβ42, NfL, and GFAP across platforms and compared performance of these markers for detecting CSF-defined A+ CU. In the Attention, Memory, and Aging Study at Stanford (AMASS) cohort with plasma samples analyzed on the updated NULISA CNS panel, we compared performance of brain-derived and total forms of pTau217, pTau181, and pTau231 for detecting amyloid PET-defined A+ CU. Finally, we conducted exploratory differential expression analyses using the full NULISAseq CNS panel in each cohort to identify novel proteins associated with AD risk factors, including age, sex, and *APOE-*ε4 carriage, as well as AD phenotypes, including amyloid burden measured using CSF Aβ42/Aβ40 (SAMS) and FBB amyloid PET (AMASS) and tau burden measured with CSF pTau181 and tau PI-2620 PET among SAMS CU.

## Method

### Participants

#### SAMS

This study included 193 participants enrolled in the Stanford Aging and Memory Study (SAMS) [24–26]. Plasma samples were collected at baseline (*n* = 162), or during a follow-up visit (*n* = 31) conducted either ~ 3.5 (*n* = 27) or ~ 7 (*n* = 4) years after baseline enrollment. Clinical diagnosis was determined at a clinical consensus meeting by a panel of neurologists and neuropsychologists based on a comprehensive neuropsychological battery. SAMS eligibility for baseline enrollment required a consensus diagnosis of CU, a Clinical Dementia Rating (CDR) score of 0 and performance within 1.5 standard deviations of demographically-adjusted means of standardized neuropsychological assessments. Of participants that only had plasma samples available during follow up (*n* = 31), 29 participants had a CDR of 0 at their longitudinal visit, one participant had a CDR of 0.5 with a CU diagnosis, and one participant was missing CDR and clinical diagnosis. The individual missing CDR was within 1.5 standard deviations of demographically-adjusted means across standardized neuropsychological assessments at follow-up. Other eligibility criteria included normal or corrected to normal vision and hearing, right-handedness, native English speaking, and no history of neurological or psychiatric disease. Participants were not excluded due to self-reported managed medical conditions, including diabetes (*n* = 13), hypertension (*n* = 15), kidney disease (*n* = 1), or high cholesterol (*n* = 16). Study protocols were approved by the Institutional Review Board of Stanford University and written informed consent was obtained from each study participant.

#### AMASS

This study included 122 CU participants enrolled in the Attention, Memory, and Aging Study at Stanford (AMASS). Plasma samples were collected at enrollment. Participants first completed a phone screen where they completed the telehealth version of the MoCA [27]. Participants with total scores > = 26 or lower scores that were judged to be due to connectivity issues were invited to complete a full in-person neuropsychological assessment, using a battery similar to SAMS. Exclusion criteria included neuropsychological test scores 1.5 SD below demographically-adjusted means on HVLT, BVMT, Logical Memory, Trails A and Trails B; contraindication to EEG, MRI, or PET; presence of a neurological, psychiatric, or medical conditions that could affect cognition; use of medications that could alter performance, or poor vision/color perception. The CDR was not administered, and clinical consensus was not performed in this study. Study protocols were approved by the Institutional Review Board of Stanford University and written informed consent was obtained from each study participant. 

### Plasma collection and analysis

Plasma samples from 315 unique participants were included in the current study, including 193 from SAMS and 122 from AMASS. Plasma collection and storage methods were harmonized across cohorts. EDTA plasma was collected by venipuncture, centrifuged for 10 min at 2000 x g, aliquoted in polypropylene tubes, and stored at -80°C until measurement. In SAMS, plasma samples were additionally analyzed on the Lumipulse platform. Lumipulse measurements of pTau217, GFAP, and NfL were available for 193 participants, Aβ42 and Aβ40 were available for 188 participants [4], and plasma pTau181 was available for 188 participants [23] (Supplementary Fig. 1).

### NULISAseq CNS disease panel

Plasma samples were analyzed at the Stanford University Human Immune Monitoring Center in two separate batches. SAMS plasma samples were processed using the NULISAseq CNS kit (*n* = 124 targets) on 7/31/2024 and AMASS plasma samples were analyzed using the updated NULISAseq CNS kit (*n* = 131 targets) on 12/02/25. Analysis procedures were harmonized across batches and were conducted as follows. Samples were thawed at room temperature. Thawing period did not exceed 3 hours. Samples were centrifuged for 1 min at room temperature (RT) at 1000 x g if there were no bubbles in the sample plate; otherwise, centrifuged for 5 min, RT at 1258 x g to remove bubbles, and loaded on an Alamar ARGO™ system. The following steps were automated on the instrument: Following incubation with capture and detection antibodies conjugated with partially double-stranded DNA containing target-specific barcodes, solutions underwent magnetic bead-based capture, wash, release, recapture, and second round of wash processes to identify bound immunocomplexes. DNA reporter molecules were generated through ligation and quantified by Next-Generation Sequencing (NGS) using an Element AVITI sequencer (Element Biosciences).

NULISA Protein Quantification (NPQ) units were used in subsequent analyses. NPQ units are target counts that were normalized in two steps and then log2-transformed. For normalization, counts were divided by internal control counts within each sample well and then subsequently divided by target-specific medians of sample replicates on each plate. The average coefficient of variation (CV) of the triplicate sample control measures was 6.0% for the first batch, and 5.5% for the second batch.

All analyses across these two NULISAseq batches were run separately and are shown side by side to guide comparisons across SAMS (batch 1) and AMASS (batch 2). The NULISAseq APOE4 target was used only for *APOE*-ε4 carrier status and was excluded from differential expression analyses leveraging the full CNS panel for both cohorts.

### Lumipulse plasma analysis

Lumipulse plasma analyses for SAMS were completed by the Stanford ADRC Biomarker Core and included pTau181 (May 2021) [23], Aβ42 and Aβ40 (May 2023) [4], and pTau217, GFAP, and NfL (December 2024). For each analysis, plasma samples were thawed on wet ice, centrifuged for 5 min at 4°C at 1000 x g, and loaded on a Lumipulse *G* 1200 instrument (Fujirebio US, Malvern, PA) as previously described [23]. Given the fully-automated nature of the Lumipulse instrument, duplicate measures are expected to yield near-identical results. Accordingly, samples were measured in singlicate within each batch.

### CSF collection and analysis

CSF data were included for 132 SAMS participants. The CSF draw took place within 1 year of their plasma draw (mean (SD) delay = 0.19 (0.08) years). CSF was collected by lumbar puncture and stored in polypropylene tubes. CSF samples were centrifuged, aliquoted, and stored at -80°C until measurement. CSF AD biomarkers Aβ42, Aβ40, and pTau181 were measured using the fully-automated Lumipulse *G* 1200 instrument as previously described [28]. CSF amyloid and tau positivity cut-offs were defined using the SAMS baseline sample [25] (*n* = 153, Supplementary Fig. 2A). CSF amyloid positivity (A+) was defined as Aβ42/Aβ40 < 0.0752, corresponding to the 0.5 probability of belonging to the positive distribution using Gaussian mixture-modelling [25]. CSF tau positivity (T+) was defined as pTau181 > 50.77, corresponding to 2SD above the mean of the A-group.

### Amyloid and tau PET imaging

Amyloid PET measured using 18F-florbetaben (FBB) was included for 120 AMASS participants with a PET scan within 3 years of their plasma draw (mean (SD) delay = 0.83 (0.826) years), with most scans (*n* = 109) occurring within 2 years of the plasma draw. Amyloid PET data were not available for two AMASS participants that withdrew from PET due to claustrophobia. Tau PET measured using 18F-PI-2620 was included for 71 SAMS participants with a tau PET scan collected within 2 years of their plasma draw (mean (SD) delay = 0.42 (0.544) years). Amyloid and tau PET scanning was completed at the Richard M. Lucas Center for Imaging at Stanford University using a PET/MRI scanner (Signa 3T, GE Healthcare). For amyloid FBB PET, emission data were collected between 90 and 110 min. For tau 18F-PI-2620 PET, emission data were collected between either 45–75 min or 60–90 min (60-90-minute data were interpolated to produce 45-75-minute images [29]). PET images were reconstructed using TOF optimized subset expectation maximization (TOF-OSEM) with 3 iterations, 28 subsets, and 2.78 × 1.17 × 1.17 mm voxel size. Corrections were applied for detector deadtime, scatter, randoms, detector normalization, and radioisotope decay. MR attenuation correction was performed with ZTE MR imaging [30]. PET data were reconstructed into 5-minute frames, and these frames were realigned and summed. Native space FreeSurfer labels defined on each participant’s T1-weighted MPRAGE structural MRI were used to extract intensity values from the co-registered summed PET data and used to create standardized uptake value ratios (SUVRs). Amyloid PET SUVRs were created for a global cortical ROI (an average across frontal, parietal, lateral temporal, and cingulate) using a whole cerebellum reference region and translated to centiloids (CL) [31]. A threshold of > 25 CL was used for amyloid positivity [4] (Supplementary Fig. 2B). Tau PET SUVR was computed using the inferior cerebellum as a reference region for a temporal cortex meta-ROI comprised of entorhinal cortex, amygdala, parahippocampal, fusiform, inferior temporal, and middle temporal gyri [32].

### *APOE* genotype

In the SAMS cohort, *APOE* genotype was determined from whole-genome sequencing (WGS) or obtained from National Cell Repository for Alzheimer’s Disease (NCRAD) using a Fluidigm fingerprint panel. *APOE* genotype (ε2/ ε3/ ε4) was determined using allelic combinations of single nucleotide variants rs7412 and rs429358. In the AMASS cohort, *APOE*-ε4 carriage was determined using the NULISAseq APOE4 target. This measure has been validated in past work leveraging NULISA in a large (*N* = 3,892) mixed disease cohort where it exhibited robust concordance (99.0%) with direct SNP genotyping [20]. Moreover, we observed 100% concordance between *APOE*-ε4 carriage determined with genotyping and the NULISAseq APOE4 measure in the SAMS cohort (Supplementary Table 1).

### Statistical analysis

All statistical analyses were conducted using R software version 4.3.0. Spearman rank correlation was used to assess the strength and direction of associations between plasma proteins measured using NULISAseq and Lumipulse assays, between NULISAseq BD-pTau and total pTau measures, and between plasma proteins and amyloid burden (CSF Aβ42/Aβ40, amyloid PET). Plots also show a regression fit and 95% confidence interval (CI). Receiver operating characteristic (ROC) curve analysis was performed to summarize the ability of individual proteins and protein ratios to differentiate amyloid status defined by CSF Aβ42/Aβ40 (SAMS) and amyloid FBB PET (AMASS). Performance of individual assays was also compared to a basic model including age, sex, and *APOE*-ε4 carriage. DeLong’s test was used to compare ROC AUC (area under the curve) across models. The Youden method was used to determine the optimal plasma cut-off to maximize sensitivity and specificity in discriminating A+ from A- individuals for each assay.

Linear regression models were used to evaluate associations between NULISA CNS targets and variables of interest, including (a) AD risk factors: age, sex, *APOE*-ε4, (b) gold standard A and T biomarkers: CSF Aβ42/Aβ40, amyloid FBB PET CL, CSF pTau181, and tau PI-2620 PET SUVR, and (c) CSF Aβ42/Aβ40-defined A status, amyloid PET-defined A status, and CSF pTau181-defined T status. Age at blood collection and sex were included as covariates in all models. All NULISA CNS proteins were in log2-transformed NPQ units, and the β coefficients from linear regression represent log2 fold-change. P-values were adjusted using the Benjamini–Hochberg (BH) method [33] to control the false discovery rate (FDR). Proteins with an FDR-adjusted *p-*value of < 0.05 were considered statistically significant. Proteins with an unadjusted *p*-value of < 0.05 were considered nominally significant. Volcano plots of log2(fold-change) against -log10(*p*-value) were used to display associations between variables of interest and NULISA CNS targets [Batch 1 (SAMS): *N* = 123 targets; Batch 2 (AMASS): *N* = 130 targets], with significant and nominally significant proteins indicated and differentiated by color.

## Results

### Participant characteristics

This study included data from 315 participants across two independent clinically unimpaired aging cohorts (Table 1). The SAMS cohort included 193 participants (mean (SD) age: 69.9 (6.35) years, 56.8% women, 86.0% non-Hispanic White). Of SAMS participants with available CSF data (*n =* 132), 27.3% (*n* = 36) were A+ and 15.9% (*n* = 21) were T+. The AMASS cohort included 122 participants (mean (SD) age: 73.1 (4.07) years, 61.5% women, 77.9% non-Hispanic White), of which 18.0% (*n* = 22) were A+ by amyloid PET. The AMASS cohort was significantly older than the SAMS cohort (*t*(312.94) = 5.52, *p* < 0.001) and did not differ in proportion of females (*X*^2^ = 0.72, *p* = 0.396) or *APOE*-ε4 carriers (*X*^2^ = 0.002, *p* = 0.965).

### Head-to-head comparison of NULISAseq and Lumipulse plasma assays

**Fig. 1:**
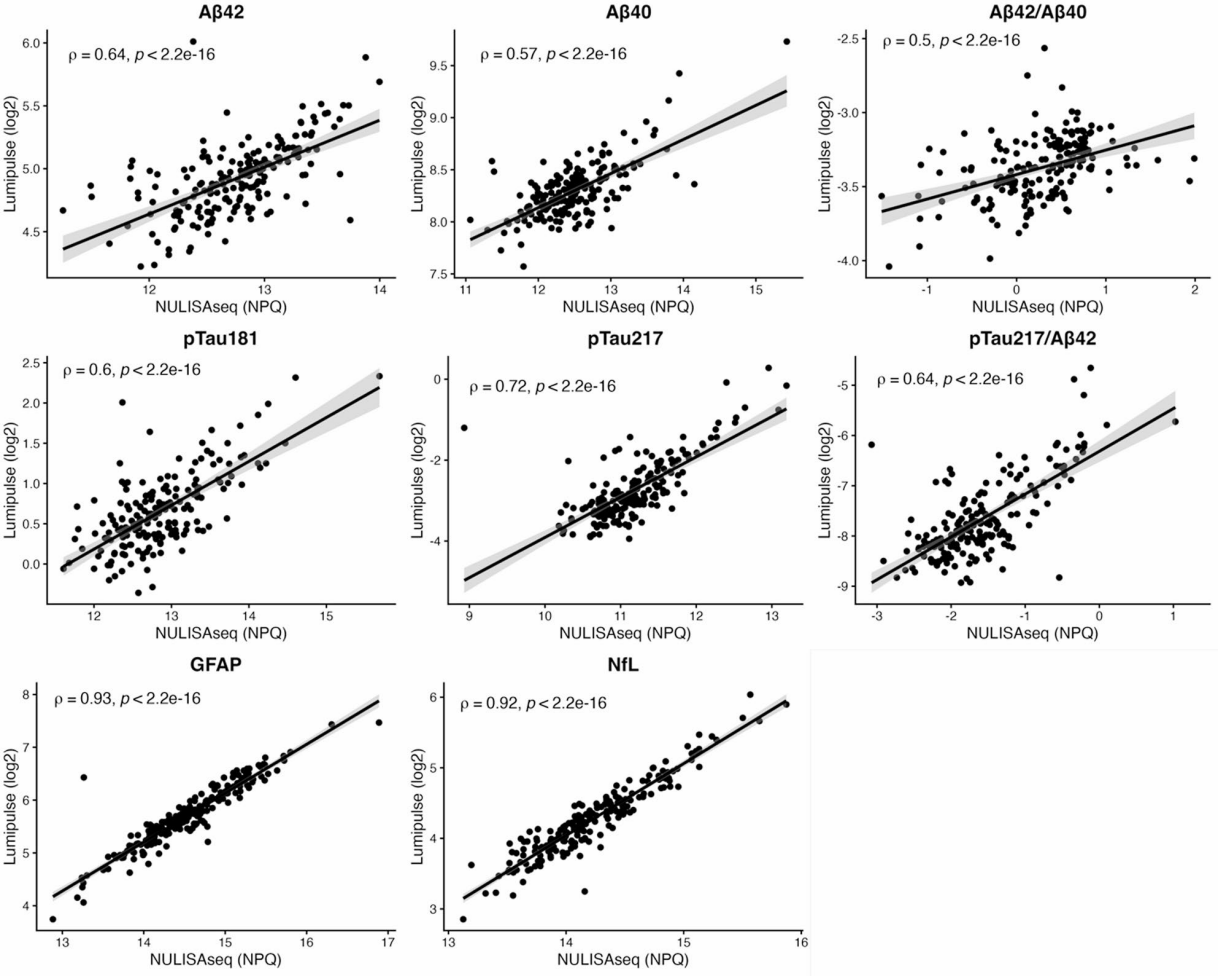
Correlations between NULISAseq and Lumipulse plasma assays. Scatterplots illustrate the distributions and correlations of proteins and protein ratios measured using the NULISA and Lumipulse platforms (pTau217, GFAP, NfL: *n* = 193; Aβ42, Aβ40, pTau181: *n* = 188). NULISAseq proteins are in NPQ (NULISA protein quantification) units and Lumipulse proteins measured in pg/mL were log2-transformed for comparison to NULISAseq. Scatterplots show the Spearman (*rho*) correlation coefficient and *p*-value, together with the best fit line and 95% confidence band

Using data from the SAMS cohort, we first examined the correlation between NULISAseq measurements and Lumipulse measurements of core AD-related plasma proteins, including Aβ42, Aβ40, pTau217, pTau181, GFAP, and NfL. We also examined plasma Aβ42/Aβ40 and pTau217/Aβ42 ratios across platforms. All pairwise comparisons exhibited moderate to high correlations, with Spearman correlation coefficients (*rho*) ranging from 0.50 to 0.93 (Fig. 1). The strongest correlations were observed for GFAP (*rho =* 0.93) and NfL (*rho =* 0.92). The weakest association was observed for the Aβ42/Aβ40 ratio (*rho =* 0.50). The correspondence across platforms between pTau measures was moderately high (*rho* values ranging between 0.60 and 0.72).

**Table 1:** Characteristics of study participants

	SAMS CU (*n* = 193)	AMASS CU (*n* = 122)
**Age, mean (SD) years**	69.9 (6.35)	73.1 (4.07)
**Sex**, ***n* (%) female**	108 (56.0%)	75 (61.5%)
**Education**,** mean (SD) years**	16.8 (2.04)	17.7 (2.34)
***APOE-*ε4**, ***n* (%) carrier**	46 (23.8%)	28 (23.0%)
***APOE*** **genotype**^**a**^		
ε2/ε3	16 (8.3%)	
ε2/ε4	3 (1.6%)	
ε3/ε3	131 (67.9%)	
ε3/ε4	39 (20.2%)	
ε4/ε4	4 (2.1%)	
**Amyloid Status**^**b**^ (*n* A+ / *n *total)	36/132 (27.3%)	22/120 (18.3%)
**CSF Aβ42/Aβ40**,** mean (SD)**	0.0877 (0.0232)	
**CSF pTau181**,** mean (SD)**	39.5 (22.1)	
**Amyloid PET CL**,** mean (SD)**		16.3 (25.1)
**Race, ** ***n*** ** (%)**		
Asian	15 (7.8%)	11 (9.0%)
Black or African American	4 (2.1%)	0 (0%)
White	166 (86.0%)	95 (77.9%)
More Than One Race	5 (2.6%)	3 (2.5%)
Other	1 (0.5%)	5 (4.1%)
Unknown/Not Reported	2 (1.0%)	3 (2.5%)
**Ethnicity, ** ***n*** ** (%)**		
Hispanic	9 (4.7%)	10 (8.2%)
NOT Hispanic	184 (95.3%)	109 (83.6%)
Unknown/Not Reported	0 (0%)	3 (2.5%)

^a^ *APOE* genotype was available only for the SAMS cohort. ^b^ Amyloid status determined by CSF Aβ42/Aβ40 (A+ < 0.0752) in SAMS and amyloid PET (A+ > 25 centiloids) in AMASS

### Performance of NULISAseq and Lumipulse plasma assays for detecting amyloid positivity

We next examined associations between CSF Aβ42/Aβ40 and both NULISA and Lumipulse immunoassays in SAMS (Supplementary Fig. 3). Across NULISAseq measurements, absolute Spearman correlations were lowest for NfL (*rho = -*0.12) and GFAP (*rho =* -0.29) and highest for pTau217/Aβ42 (*rho =* -0.55) and pTau217 (*rho* = -0.50). Correlations exhibited a similar pattern across Lumipulse assays, with absolute Spearman correlations that were lowest for NfL (*rho = -*0.15) and GFAP (*rho =* -0.28) and highest for pTau217/Aβ42 (*rho =* -0.59), pTau217 (*rho* = − 0.53), and Aβ42/Aβ40 (*rho* = 0.56).

**Fig. 2:**
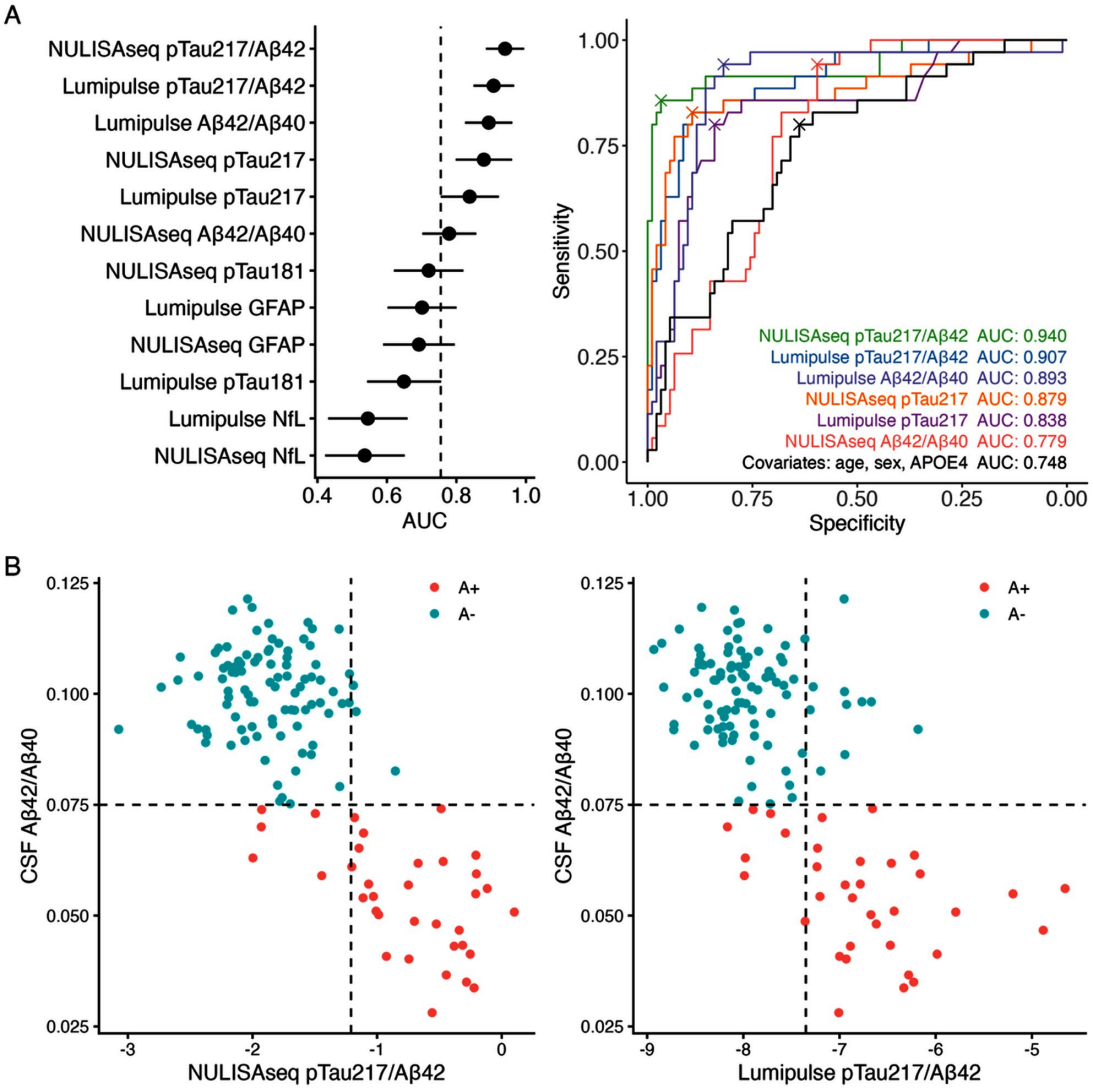
Performance of NULISAseq and Lumipulse plasma assays for detecting CSF amyloid positivity. **A** Forest plot depicting AUC and 95% CI for individual assays and biomarker ratios in detecting CSF-defined A+ CU (left). The dotted line indicates the AUC of a covariate-only model including age, sex, and *APOE-*ε4. Corresponding ROC curves for the top six performing models across platforms and the covariate-only model are plotted (right). **B** Concordance between CSF Aβ42/Aβ40 and plasma pTau217/Aβ42 measured using NULISAseq and Lumipulse (*n* = 129). Dotted lines indicate the amyloid positivity cut-offs for each modality, giving rise to four participant profiles

Using ROC curve analyses, we compared the performance of NULISAseq and Lumipulse immunoassays for detecting CSF A+ in SAMS CU (*n* = 129), examining individual proteins pTau217, pTau181, GFAP, and NfL as well as plasma protein ratios Aβ42/Aβ40 and pTau217/Aβ42 (Fig. 2A; Supplementary Table 2). Across platforms, plasma pTau217/Aβ42 exhibited the highest performance in discriminating CSF-defined amyloid positivity in CU, with an AUC of 0.940 (95% CI: 0.885–0.995) for NULISAseq and an AUC of 0.907 (95% CI: 0.849–0.966) for Lumipulse. The performance of these two assays did not significantly differ (Z = 1.56, *p* = 0.119). The pTau217/Aβ42 ratio significantly outperformed pTau217 alone for both NULISAseq (AUC: 0.879, 95% CI: 0.798, 0.960; Z = 2.22, *p* = 0.026) and Lumipulse (AUC: 0.838, 95% CI: 0.755, 0.922; Z = 3.60, *p* = 0.0003) assays, with NULISAseq pTau217 marginally outperforming Lumipulse pTau217 (Z = 1.66, *p* = 0.097). In contrast, Lumipulse Aβ42/Aβ40 (AUC: 0.893, 95% CI: 0.825, 0.961) significantly outperformed NULISAseq Aβ42/Aβ40 (AUC: 0.779, 95% CI: 0.702, 0.857; Z = 2.66, *p* = 0.008), with NULISAseq Aβ42/Aβ40 not significantly outperforming a covariate-only model (*Z* = 0.55, *p* = 0.582). The performance of pTau181, GFAP, and NfL did not differ across platforms (all *p* > 0.10). None of these biomarkers performed better than a covariate only-model for detecting CSF A+, with NfL performing significantly worse than the covariate-only model (NULISAseq: *Z* = 3.89, *p* < 0.001; Lumipulse: *Z* = 3.70, *p <* 0.001), consistent with weak correlations between CSF Aβ42/Aβ40 and NfL across platforms (Supplementary Fig. 3).

We next selected the highest performing assay across platforms, pTau217/Aβ42, and derived a plasma threshold for predicting CSF A+, using the Youden method to maximize sensitivity and specificity. We observed strong agreement across modalities using the NULISAseq pTau217/Aβ42-defined positivity threshold (Fig. 2B, left), such that 93.8% of participants (*n* = 121) had concordant plasma and CSF profiles and 6.2% (*n* = 8) had discordant profiles. We also observed strong agreement across modalities with the Lumipulse pTau217/Aβ42-derived positivity threshold (Fig. 2B, right), with 87.6% of participants (*n =* 113) exhibiting concordant plasma and CSF profiles, however twice as many participants (12.4%, *n* = 16) exhibited discordant profiles compared to that observed with the NULISAseq ratio.

### Performance of brain-derived plasma pTau assays for detecting amyloid positivity

In the AMASS cohort, we leveraged the updated NULISA CNS panel to compare the performance of brain-derived and total assays of pTau217, pTau181, and pTau231, as well as other relevant AD markers pTau217/Aβ42, Aβ42/Aβ40, GFAP, and NfL. First, we examined associations between brain-derived and total pTau concentrations (Supplementary Fig. 4), which revealed moderate to high correlations ranging from *rho* = 0.60 for pTau231, *rho* = 0.72 for pTau181, and *rho* = 0.80 for pTau217. Next, we characterized associations between these six pTau assays and amyloid PET CL (Supplementary Fig. 5). The strongest associations were observed for BD-pTau181 (*rho* = 0.46) and BD-pTau217 (*rho* = 0.45), with the weakest association observed for total-pTau181 (*rho* = 0.34).

**Fig. 3:**
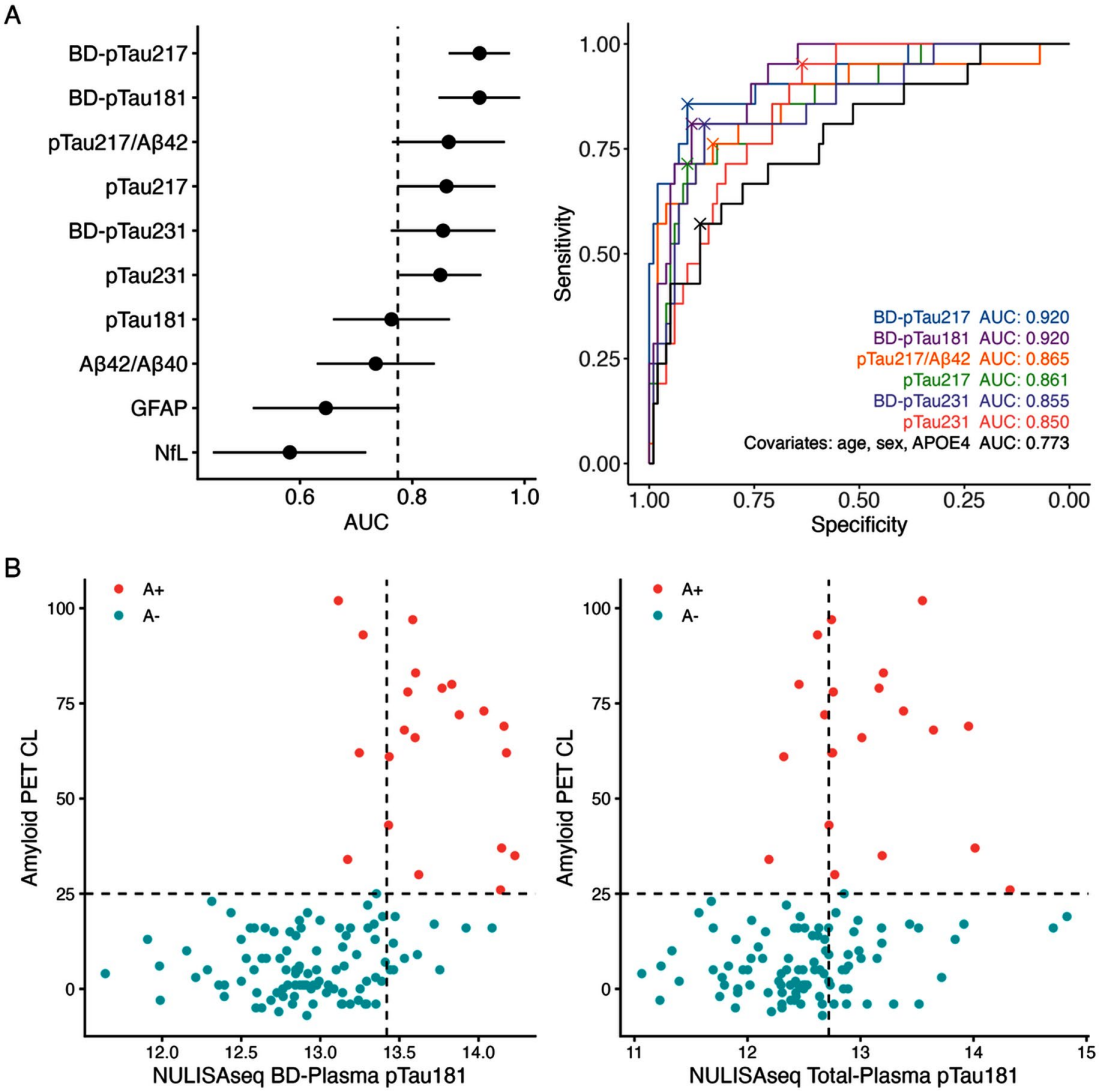
Performance of NULISAseq brain-derived and total pTau plasma assays for detecting amyloid PET positivity. **A** Forest plot depicting AUC and 95% CI for individual assays and biomarker ratios in detecting amyloid PET positivity (left). The dotted line indicates the AUC of a covariate-only model including age, sex, and *APOE-*ε4. Corresponding ROC curves for the top six performing models and the covariate-only model (right). **B** Concordance between amyloid PET centiloid (CL) and BD-plasma pTau181 (left) and total-plasma pTau181 (right; *n* = 120). Dotted lines indicate the amyloid positivity cut-offs for each modality, giving rise to four participant profiles

To evaluate the discriminative accuracy of BD and total pTau isoforms for detecting amyloid PET positivity in AMASS CU (*n* = 120), we conducted an ROC curve analysis (Fig. 3A; Supplementary Table 3). BD-pTau217 and BD-pTau181 exhibited the highest performance, with AUCs of 0.920 (95% CI: 0.849–0.992) and 0.920 (0.865–0.974), respectively. Performance of these BD isoforms was marginally higher than total plasma measurements for pTau217 (AUC: 0.861, 95% CI: 0.774–0.949; Z = 1.76, *p* = 0.078) and significantly higher for pTau181 (AUC: 0.763, 95% CI: 0.659–0.867; Z = 3.21, *p* = 0.001). In contrast, BD-pTau231 (AUC: 0.855, 95% CI: 0.762–0.948) performed comparably to total pTau231 (AUC: 0.850, 95% CI: 0.777–0.924; Z = 0.107, *p* = 0.915). The pTau217/Aβ42 ratio had an AUC of 0.865 (95% CI: 0.764–0.965), performing similarly to pTau217 alone and qualitatively worse though not statistically different from BD-pTau217 (Z = 1.5491, *p* = 0.124). Across biomarkers, only BD-pTau217 (Z = 2.84, *p* = 0.005) and BD-pTau181 (Z = 2.28, *p* = 0.023) significantly outperformed the covariate-only model (AUC: 0.773, 95% CI 0.657–0.889; all other *p* > 0.108), while Aβ42/Aβ40 performed similarly (AUC: 0.735, 95% CI: 0.630–0.840, *p* = 0.609) and GFAP (AUC: 0.646, 95% CI: 0.516–0.777; *p* = 0.061) and NfL (AUC: 0.582, 95% CI: 0.445–0.718; *Z* = 2.04, *p* = 0.041) tended to perform worse.

We next derived thresholds to maximize sensitivity and specificity for each plasma pTau target and examined concordance between plasma-defined and PET-defined A status. For BD-pTau181, concordant plasma and PET profiles were observed among 89.2% of participants (*n* = 107) while 10.8% (*n* = 13) had discordant profiles (Fig. 3B). For total-pTau181, concordant plasma and PET profiles were observed among 72.5% of participants (*n* = 87), with over twice as many participants exhibiting discordant profiles (27.5%; *n* = 33). Interestingly, most of these discordant cases were false positives (*n* = 28; i.e., above threshold for pTau181 and A- on amyloid PET). Similarly, agreement varied substantially between BD-pTau231 and total-pTau231, with 85% of participants (*n* = 102) compared to 69.2% of participants (*n =* 86) exhibiting concordant profiles, respectively. Again, discordant cases for total-pTau231 (*n =* 37) were driven by a substantial increase in false positives (*n =* 36) compared to BD-pTau231 (*n =* 14). The difference in agreement with amyloid PET status across BD- and total-assays was more modest for pTau217, with 89.2% (*n =* 107) agreement for BD-pTau217 and 87.5% (*n =* 105) agreement for total-pTau217. Together, these findings suggest that improvements in agreement with amyloid PET for BD-pTau assays compared to total-pTau assays can be substantial, and that the magnitude of this improvement may differ across pTau isoforms.

### Association of NULISAseq CNS panel targets with age, sex, and *APOE-*ε4

**Fig. 4:**
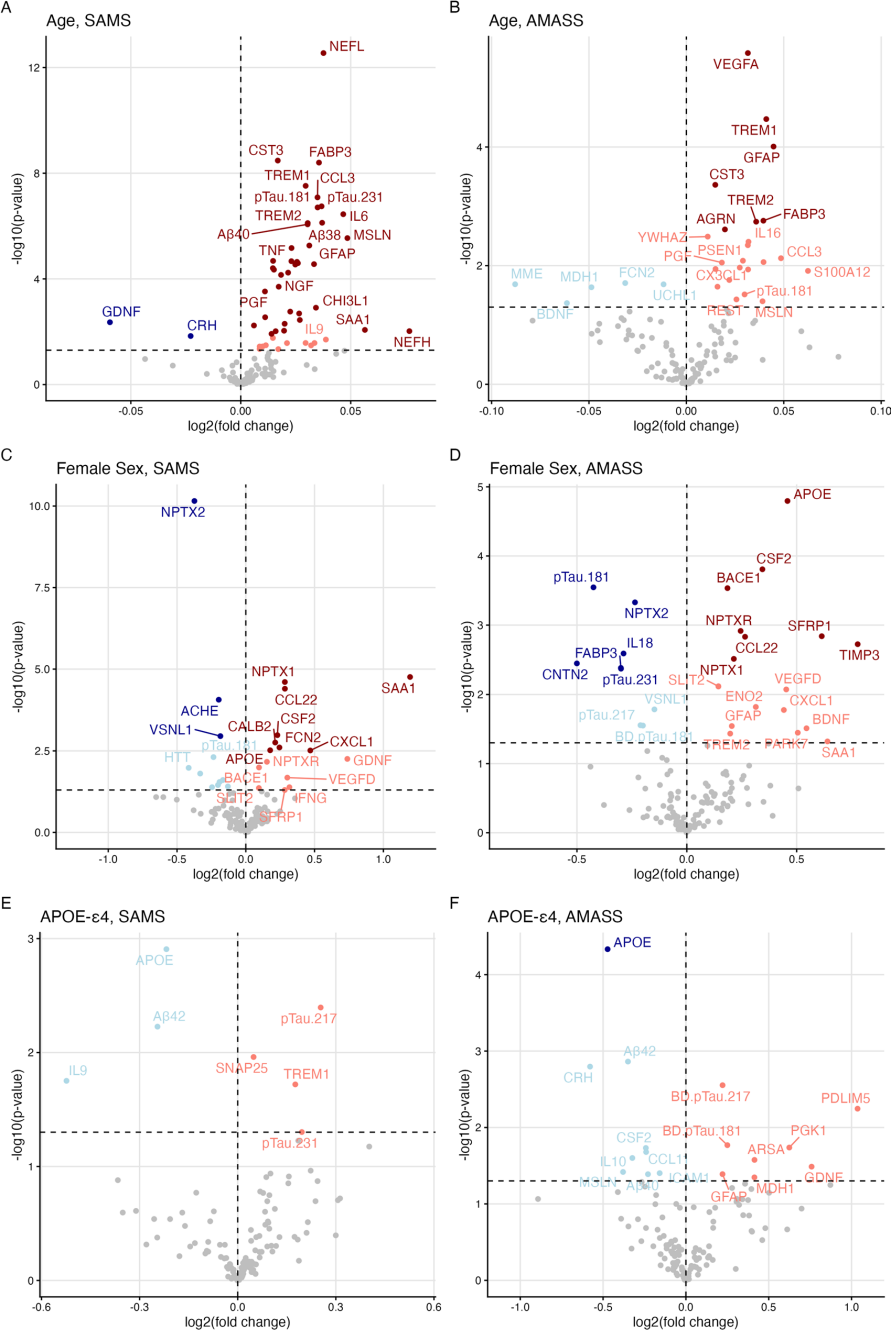
Association of NULISAseq plasma proteins with age, sex, and *APOE*-ε4. **A-F** Volcano plots display proteins differentially expressed as a function of age, sex, and *APOE*-ε4 in SAMS CU (*n* = 193) across 123 CNS proteins and AMASS CU (*n* = 122) across 130 CNS proteins. The x-axis represents the log2(fold change) and the y-axis depicts -log10 of the raw *p*-value. Significant targets, defined by an FDR-adjusted *p*-value of < 0.05, are shown in red (upregulated) or blue (downregulated). Light-colored points indicate targets with nominally significant differences (unadjusted *p*-value < 0.05). Grey points indicate non-significant targets

We next conducted exploratory analyses with the full NULISAseq CNS panel to identify proteomic abundance patterns associated with common AD risk factors, including age, sex, and *APOE-*ε4 carriage across our two independent CU cohorts (Fig. 4). In SAMS, a total of 40 targets (33% of CNS proteins) exhibited significant associations with age, controlling for sex (Fig. 4A, Supplementary Fig. 6A). These included proteins implicated in neurodegeneration and axonal injury, including NFL and neurofilament heavy polypeptides (NEFH), fatty acid-binding protein 3 (FABP3), cystatin C (CST3), and agrin (AGRN), as well as proteins related to AD, including pTau231, pTau181, and pTau217, MAPT, Aβ40, Aβ38. Also associated with age were proteins relating to microglial activation and inflammation, including triggering receptor expressed on myeloid cells 1 (TREM1) and 2 (TREM2), GFAP, chitinase-3-like protein 1 (CHI3L1/YKL-40), serum amyloid A1 (SAA1), along with several cytokines (IL6, IL33, IL16) and chemokines (CCL4, CCL3, CCL11, CXCL10), and vascular endothelial growth factor A (VEGF-A), a protein important for maintaining cerebrovascular integrity. While most proteins were upregulated with age, glial cell line-derived neurotrophic factor (GDNF) and corticotropin-releasing hormone (CRH) were downregulated with age. Among AMASS CU, age was significantly associated with upregulation of 7 targets (5% of CNS proteins), replicating those identified in the SAMS cohort (Fig. 4B, Supplementary Fig. 6B). These included GFAP, TREM1, TREM2, FABP3, AGRN, CST3, and VEGF-A, highlighting age-related upregulation of proteins related to neuronal injury, microglial activation, and cerebrovascular integrity.

Sex differences were observed across 11 targets (8.9% of CNS proteins) among SAMS CU (Fig. 4C, Supplementary Fig. 6C). Proteins elevated in females included several targets involved in the innate immune response, including SAA1, APOE, ficolin-2 (FCN2), chemokines CCL22 and CXCL1, the cytokine colony-stimulating factor 2 (CSF2), as well as neuronal pentraxin 1 (NPTX1) and calretinin (CALB2). Proteins lower in females compared to males included neuronal pentraxin 2 (NPTX2), acetylcholinesterase (ACHE), and visinin like 1 (VSNL1). Among AMASS CU, sex differences were observed in 14 targets (10.8% of CNS proteins; Fig. 4D, Supplementary Fig. 6D). Five of these targets replicated those identified in SAMS, including upregulation of APOE, NPTX1, CSF2, and CCL22 and downregulation of NPTX2 in females compared to males. This cohort additionally exhibited higher expression of tissue inhibitor of metalloproteinases-3 (TIMP3), secreted frizzled-related protein 1 (SFRP1), neuronal pentraxin receptor (NPTXR), and beta-secretase 1 (BACE1) in females. Proteins that exhibited downregulation in females compared to males included pTau181 and pTau231, FABP3, IL18, and contactin-2 (CNTN2), a neuronal cell-adhesion molecule linked to neuronal integrity.

Associations between *APOE-*ε4 carriage and CNS proteins, controlling for age and sex, did not survive the FDR-adjusted significance threshold in either cohort, with the exception of downregulation of APOE in ε4 carriers compared to non-carriers in AMASS CU (Fig. 4E and F). However, nominally significant targets that replicated across study populations included downregulation of Aβ42 and APOE and upregulation of pTau isoforms in ε4 carriers compared to non-carriers.

### Association of NULISAseq CNS panel targets with amyloid biomarkers

**Fig. 5:**
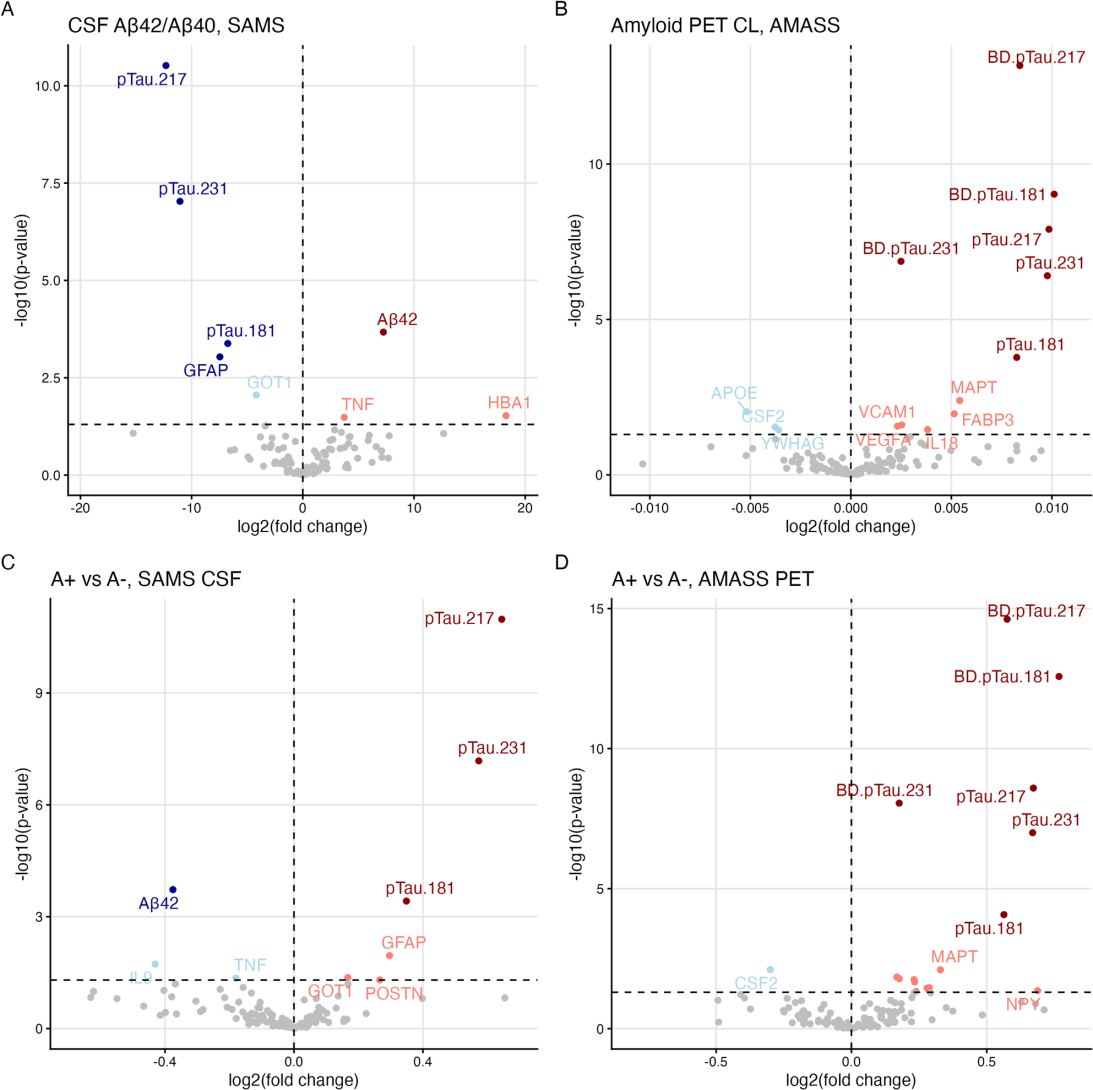
Association of NULISAseq plasma proteins with amyloid biomarkers. **A-B** Volcano plots display proteins differentially expressed as a function of amyloid burden measured using CSF Aβ42/Aβ40 in SAMS (*n* = 132) and amyloid PET in AMASS (*n* = 120) and **C-D** amyloid status in SAMS (A-, *n* = 96, A+, *n* = 36) and AMASS (A-, *n* = 98, A+, *n* = 22). The x-axis represents the log2(fold change) and the y-axis depicts -log10 of the raw *p*-value. Significant targets, defined by an FDR-adjusted *p*-value of < 0.05, are shown in red (upregulated) or blue (downregulated). Light-colored points indicate targets with nominally significant differences (unadjusted *p*-value < 0.05). Grey points indicate non-significant targets

We next conducted an exploratory analysis to identify NULISAseq CNS panel proteins associated with amyloid burden across cohorts, measured using CSF Aβ42/Aβ40 in SAMS and amyloid PET in AMASS (Fig. 5). Controlling for age and sex, we found that abnormal CSF Aβ42/Aβ40 was associated with upregulation of plasma pTau217, pTau231, pTau181, and GFAP and downregulation of Aβ42 (Fig. 5A). Similarly, amyloid PET burden (CL) was associated with upregulation of brain-derived and total forms of pTau217, pTau181, and pTau231 (Fig. 5B). These findings underscore a strong link between the development of amyloidosis in CU and differential expression of pTau proteins, while also suggesting early involvement of astrocytic activation. To further probe relationships between abnormal amyloid and CNS proteins, we next examined differences in NULISAseq proteins as a function of amyloid status (A+ vs. A-) controlling for age and sex in each cohort (Fig. 5CD). In SAMS, A+ individuals exhibited average fold increases of 56.3%, 48.8%, and 27.4% in pTau217, pTau231 and pTau181, respectively, and a 22.9% decrease in Aβ42 compared to A- (Supplementary Fig. 7A). Among AMASS CU, A+ individuals exhibited the largest fold change differences in BD-pTau181 at 70.2%, with fold increases of 59.4% for total-pTau217, 59.4% for total-pTau231, 49.0% for BD-pTau217, 47.8% for total-pTau181, and 13.0% for BD-pTau231 (Supplementary Fig. 7B).

### Association of NULISAseq CNS panel targets with tau biomarkers

**Fig. 6:**
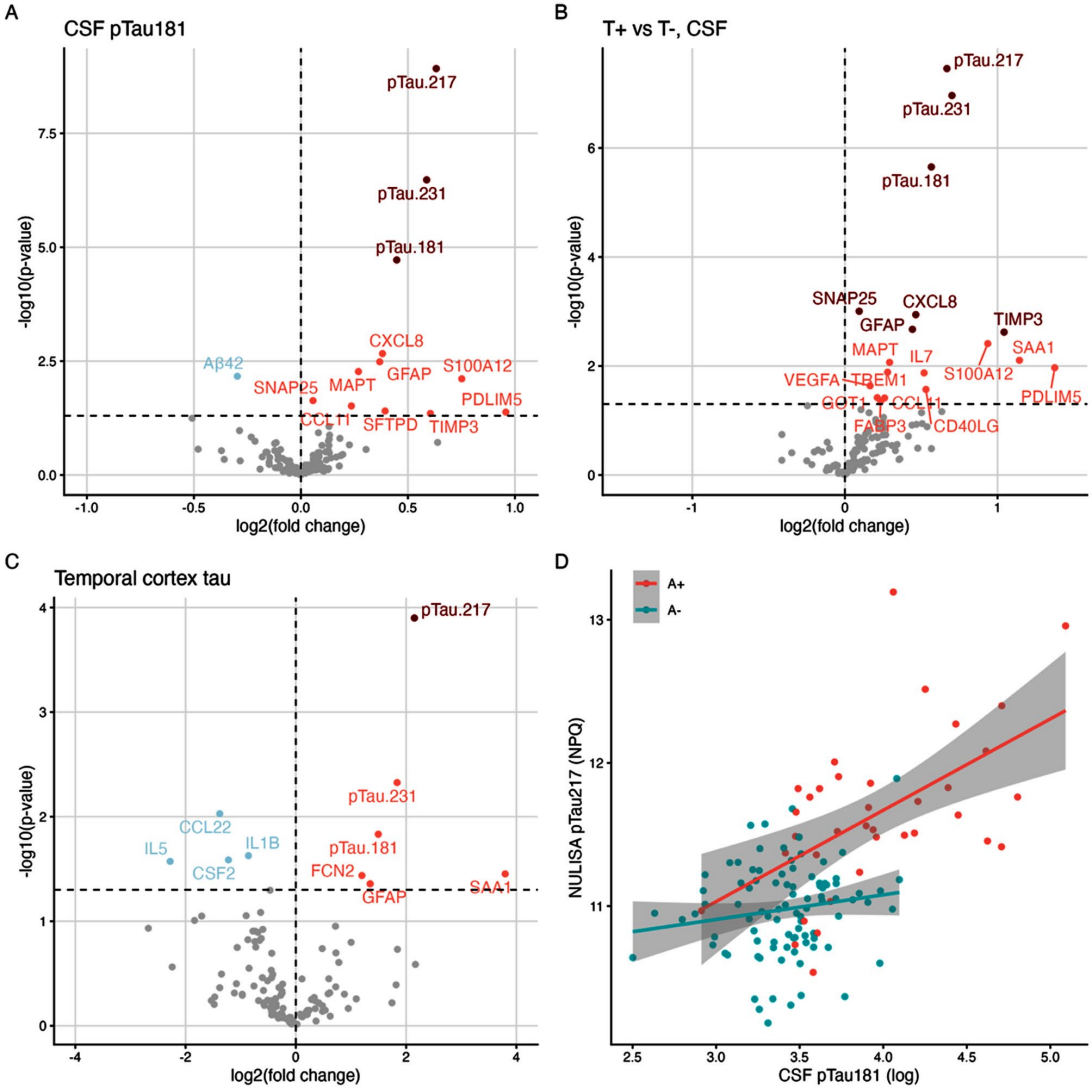
Association of NULISAseq plasma proteins with tau biomarkers. **A-C** Volcano plots illustrate proteins differentially expressed as a function of CSF pTau181 (*n* = 132), CSF-defined tau status (T-, *n* = 111; T+, *n* = 21) and temporal cortex tau SUVR (*n* = 71) in SAMS CU. The x-axis represents the log2(fold change) and the y-axis depicts -log10 of the raw *p*-value. Significant targets, defined by an FDR-adjusted *p*-value of *<* 0.05, are shown in red (upregulated) or blue (downregulated). Light-colored points indicate targets with nominally significant differences (unadjusted *p*-value < 0.05). Grey points indicate non-significant targets. **D** Scatterplot illustrates the relationship between CSF pTau181 (log transformed) and NULISAseq plasma pTau217 by amyloid status (A-, *n* = 96; A+, *n* = 36). The best fit line and 95% confidence band are plotted for each group

We next conducted an exploratory analysis to identify NULISAseq CNS proteins associated with tau burden in the SAMS cohort, assayed using CSF pTau181 and focal tau accumulation in temporal cortex measured using tau PET imaging. As expected, these tau measures were moderately correlated with each other in the subsample of individuals with both measures available (*rho* = 0.37, *p* = 0.045, *n* = 30; Supplementary Fig. 11A); given the small sample size, consideration of this effect size estimate should be treated with caution. Differential expression analyses controlling for age and sex revealed upregulation of pTau217, pTau231, pTau181 with abnormal CSF pTau181 (Fig. 6A). When CSF Aβ42/Aβ40 was additionally included as a covariate, pTau217 remained significantly upregulated (fold change = 1.31, *p*_fdr_ *=* 0.038). When participants were stratified into T+ and T- groups based on CSF pTau181 (Fig. 6B, Supplementary Fig. 7C), the T+ group exhibited fold-change increases of 62.6%, 58.9%, and 48.1% for pTau231, pTau217, and pTau181, respectively. T+ CU additionally exhibited significant upregulation of proteins related to innate immune activation, including a 1.38-fold increase in pro-inflammatory chemokine CXCL8 (95% CI: 1.14, 1.67), a 1.36-fold increase in GFAP (95% CI: 1.12, 1.65), a 2.06-fold increase in TIMP3 (95% CI: 1.30, 3.27), and a 1.07-fold increase in SNAP-25 (95% CI: 1.03, 1.11). The increased presence of these proteins linked to altered inflammatory signaling suggests that tau-associated inflammatory processes are initiated early, as demonstrated in these CU individuals. In SAMS participants with tau PET data (*n* = 71, Supplementary Table 4), we observed significant upregulation of plasma pTau217 (fold change = 4.44, *p*_fdr_ *=* 0.015; Fig. 6C, Supplementary Fig. 11B). Together with the CSF findings described above, these results suggest that upregulation of plasma pTau217 is linked to both abnormal amyloid and tau accumulation.

Given observed associations between measures of both amyloid and tau burden with plasma pTau217, we sought to characterize relationships between all three measures (Fig. 6D). To this end, we examined the association between CSF pTau181, as a marker of tau progression, with plasma pTau217, and whether this association was modified by CSF-defined amyloid status. This revealed independent main effects of amyloid status (β = 0.46, *p* < 0.001) and CSF pTau181 (β = 0.37, *p* < 0.001) on pTau217 and a significant amyloid status by CSF pTau181 interaction (β = 0.42, *p* = 0.036), such that plasma pTau217 was elevated in A+ compared to A- CU, even at lower levels of CSF pTau181. Importantly, plasma pTau217 was positively associated with levels of CSF pTau181 within A+ CU (β = 0.59, *p* < 0.001), suggesting that pTau217 continues to become elevated as CSF pTau181 levels increase. While cross-sectional, these findings are consistent with an initial increase in plasma pTau217 with amyloid positivity and continued rise with the progression of abnormal tau.

## Discussion

Scalable, multi-analyte plasma proteomic panels capable of accurately detecting initial AD pathology and related pathophysiological processes relevant for disease risk among CU older adults are critical for early detection and prognosis. Critically, this field is rapidly evolving, evidenced by the introduction of highly sensitive multiplex plasma proteomic panels tailored to established AD biomarkers, such as NULISA, together with the addition of novel AD-relevant targets to NULISA’s CNS panel, including assays targeting brain-derived pTau isoforms. In this study, we leveraged two independent biomarker-characterized CU cohorts to evaluate the NULISA platform in three important ways. First, we conducted a head-to-head comparison between core AD biomarkers measured using NULISA and corresponding single-plex immunoassays on the fully-automated Lumipulse platform, demonstrating moderate to high correlations across biomarkers of pTau, Aβ, NfL, and GFAP and establishing equivalent performance of NULISAseq pTau217/Aβ42 and pTau217 for detecting CSF-defined A+ as corresponding Lumipulse plasma assays. Second, we compared the performance of BD-pTau and total-pTau isoforms for detecting amyloid PET positive CU, demonstrating excellent and equivalent accuracy of BD-pTau217 and BD-pTau181, with significant performance gains compared to corresponding total pTau measures. Finally, we conducted exploratory differential abundance analyses and identified targets related to AD risk and AD phenotypes among CU, including proteins linked to neuronal injury, astrocytic activation, inflammation, and synaptic integrity. Taken together, the present results provide robust evidence that NULISAseq assays are sensitive to abnormal AD biomarkers across two independent CU cohorts and highlight the value of leveraging plasma multiplexing to characterize early abnormalities both related to and independent of core AD biomarkers in the context of preclinical AD.

Through head-to-head comparison of protein measurements from NULISA and established Lumipulse single-plex plasma immunoassays, we provide evidence for moderate to high correlations for pTau217, pTau181, Aβ42, Aβ40, NfL, and GFAP. These findings align with prior work demonstrating good correspondence between NULISAseq and ALZpath Simoa immunoassays in predominantly CU cohorts [19, 22, 34] as well a large mixed-dementia cohort comparing NULISAseq to single-plex immunoassays [20]. The current findings provide further validation of NULISAseq analyte measurements in a CU cohort. As in prior work [19, 20], correlations tended to be higher for pTau, NfL and GFAP, while those for Aβ tended to be moderate. Differences in the strength of cross-platform correlations across proteins may be due to differences in protein characteristics that alter their susceptibility to pre-analytic and analytic factors. For example, Aβ proteins are more prone to aggregation, adsorption to tube surfaces, and binding to carrier proteins, which may contribute to greater measurement variability compared to NfL and GFAP. Indeed, when comparing performance of NULISA and Lumipulse assays for detecting CSF amyloid positivity, NULISA Aβ42/Aβ40 performed worse than Lumipulse Aβ42/Aβ40 and did not reliably outperform a covariate-only model with age, sex, and *APOE-*ε4 in SAMS, nor in AMASS when detecting amyloid PET positivity. These observations across two independent cohorts suggest that the use of plasma Aβ42/Aβ40 alone as a “Core 1” biomarker of Aβ proteinopathy in biological staging frameworks for AD [35] may not be appropriate for the current NULISAseq assay.

In contrast, the present results provide novel evidence for excellent performance of NULISAseq plasma pTau217/Aβ42 (AUC 0.940) and pTau217 (AUC 0.879) in detecting CSF-defined amyloid positivity in SAMS. Critically, these biomarkers performed as well as top performing single-plex immunoassays on the Lumipulse platform, including plasma pTau217/Aβ42 (AUC 0.907), Aβ42/Aβ40 (AUC 0.893), and pTau217 (AUC 0.838). NULISAseq pTau217 and pTau217/Aβ42 also performed well in detecting amyloid PET positivity in the AMASS cohort, with AUCs of 0.86, providing evidence for robust performance across gold standard biomarkers of brain amyloidosis. These independent observations build on existing work demonstrating equivalent performance of plasma pTau217 measured using NULISAseq and Simoa immunoassays for detection of amyloid PET positivity in predominantly CU cohorts [19, 22] and a recent study demonstrating robust performance of NULISAseq pTau217 and pTau217/Aβ42 for detecting amyloid PET positivity and clinical AD status in a large mixed-dementia cohort [20]. Interestingly, we found that pTau217/Aβ42 outperformed pTau217 alone in SAMS but not in AMASS. While few studies have compared these measures directly, some prior work has observed superior diagnostic performance of pTau217/Aβ42 compared to pTau217 for detecting amyloid positivity [36, 37]. Given existing research suggesting plasma ratios are less susceptible to measurement confounds related to kidney function and other comorbidities compared to individual analytes [36, 38], the pTau217/Aβ42 ratio may be well suited for detecting amyloid positivity, particularly in real-world settings [37]. However, more research is needed to establish the value of incorporating Aβ42, particularly with the rapid evolution of available plasma pTau measures.

Indeed, motivated by observations that plasma pTau fragments can originate from both the CNS and peripheral sources, NULISA’s updated CNS panel now includes assays that specifically target CNS-released (i.e., brain-derived) pTau isoforms. This development is important, as it may help address challenges related to peripheral contributions to plasma pTau concentrations that could alter biomarker specificity, including signal related to degenerating peripheral nerves and muscle fibers in conditions such as ALS [14, 16]. Only one published study to date has reported performance of NULISAseq BD-pTau217 for detecting amyloid positivity, in which it outperformed total-pTau217 measured using Simoa [39]. The current study builds on this work by demonstrating excellent performance of BD-pTau217 and BD-pTau181 for detecting amyloid PET positivity in the AMASS cohort, with AUCs of 0.920 across isoforms. This represented a modest improvement over total pTau217 (AUC 0.86) and a significant improvement compared to total pTau181 (AUC 0.76). Interestingly, the improvement in performance for BD-pTau181 was related to a significant reduction in false positives, consistent with greater specificity of BD-pTau181 for CNS-released pTau linked to cerebral amyloid accumulation. Together, these results provide novel evidence that measurements of BD-pTau can improve accuracy in detecting amyloid positivity in CU and suggest that BD-pTau181 may provide comparable sensitivity to amyloid burden as BD-pTau217. Interestingly, while BD-pTau231 and total-pTau231 exhibited comparable performance for detecting A+ with AUCs of 0.85, BD-pTau231 yielded higher classification agreement with amyloid PET at its Youden-based threshold. While these findings require replication in independent cohorts, they provide critical initial evidence for robust performance of NULISAseq BD-pTau assays in detecting abnormal amyloid PET among CU in the rapidly evolving landscape of accessible plasma proteomic measures for detection of AD pathology.

Consistent with strong performance of NULISAseq pTau assays for detecting cerebral amyloid accumulation across cohorts, proteomic abundance analyses revealed upregulation of all pTau isoforms with amyloid burden measured using CSF or PET across cohorts. These results are consistent with the classification of these proteins as Core 1 plasma biomarkers and existing findings that phosphorylated mid-region fragments pTau217, pTau181, and pTau231 become abnormal around the same time as amyloid PET [40, 41]. Notably, we also observed upregulation of pTau217 with abnormal CSF pTau181, even after controlling for CSF Aβ42/Aβ40, as well as with temporal cortex tau accumulation. Indeed, amyloid status and CSF pTau181 explained unique variance in plasma pTau217 concentrations, such that increasing CSF pTau181 burden was associated with elevated plasma pTau217 even among A+ CU. While these results are cross-sectional, they suggest that pTau217 does not plateau following amyloid positivity but continues to exhibit increases with tau progression. These findings are consistent with past work demonstrating that pTau217 was the only plasma biomarker among Aβ42/Aβ40, pTau181, pTau231, NfL, and GFAP to exhibit continued change in A+ individuals over 4–6 years that were also linked to clinical progression and atrophy [41]. Together, these findings have implications for the utility of pTau217 as both a biomarker of the presence of abnormal amyloid and as a candidate surrogate marker of disease progression. However, NULISAseq longitudinal data linking change in plasma pTau217 to change in tau burden, cognition, and clinical status are needed to further support this possibility.

Beyond core A and T biomarkers, there is growing appreciation of the role of maladaptive inflammation and synaptic dysfunction in the pathophysiology and progression of AD [35, 42, 43]. Consistent with this view, the present study identified several markers of inflammation and synaptic integrity that were upregulated with AD phenotypes. Plasma GFAP, a measure of astrocytic activation, was significantly upregulated with abnormal CSF Aβ42/Aβ40 and pTau181 in SAMS. Rising GFAP has been linked to initial amyloid accumulation among CU and is thought to reflect astrocyte-mediated neuroinflammatory responses to amyloid deposition [44–46] that may influence the relationship between cerebral amyloid and downstream tau phosphorylation and tangle accumulation [47]. Tau positivity in SAMS was also associated with upregulation of IL-8, a pro-inflammatory chemokine produced by astrocytes and microglia that has been associated with abnormal amyloid and tau accumulation [48]. SNAP-25, a key marker of synapse degeneration that is elevated in AD [49] and has been linked to abnormal amyloid and tau biomarkers and progression to dementia [50], was also upregulated with tau positivity in SAMS. While the early data implicating these biomarkers in AD pathophysiology and progression come primarily from CSF proteomics, emerging evidence from plasma proteomics, including the present and recent work leveraging NULISA [19, 20], are converging to implicate systemic inflammation and synaptic changes in aging and AD pathophysiology. Critically, the present findings suggest that these changes in response to AD pathology begin early and can be detected among CU older adults. These results motivate future research leveraging multi-analyte proteomic panels to characterize associations between early-changing plasma markers of inflammation and synaptic integrity and progression to cognitive impairment.

Key strengths of the current study include the inclusion of two independent CU cohorts, head-to-head comparison of NULISAseq assays with established Lumipulse immunoassays, and validation of novel NULISAseq BD-pTau assays for detecting A+ among CU. This study also has limitations. Given modest rates of amyloid and tau positivity in the current CU cohorts, sample sizes for analyses examining proteomic abundance differences in biomarker positive groups are relatively small, and these findings warrant further exploration in larger samples. The aging cohorts studied here are not representative of the broader population and future work is needed to establish the performance of this NULISAseq technology in real-world settings, including vulnerability to potential confounders related to health comorbidities. Current NULISAseq methods only allow relative quantification, which precludes the identification of a single cut-off for amyloid positivity that can be applied across different batches. As this new technology continues to develop, it will be important to provide absolute quantification for key AD targets such as total or BD-pTau isoforms. Finally, these results are cross-sectional, and it will be important for future longitudinal studies to examine within-subject reliability and change in these plasma markers over time, as well as determine the prognostic value of NULISAseq proteins for predicting future cognitive decline and disease progression.

## Conclusions

Taken together, this study provides novel evidence validating NULISAseq AD biomarkers against established Lumipulse single-plex immunoassays and demonstrates robust performance of novel NULISAeq brain-derived pTau assays for detection of amyloid burden among CU. These findings are significant, given the critical need for multi-analyte panels that can simultaneously measure known AD-relevant targets using a small blood volume without compromising sensitivity. Indeed, multiplexing enabled the identification of additional targets linked to inflammation, neuronal injury, and synaptic integrity that were associated with AD risk factors and abnormal AD biomarkers. These findings highlight the potential to leverage multiplexed proteomics to support rich phenotyping and characterization of mechanisms both related to and independent of core AD biomarkers that contribute to early AD pathological change and cognitive decline in older CU populations.

## Supplementary Information

Below is the link to the electronic supplementary material.

Supplementary Material 1: Additional file 1: Supplementary Tables S1-S4 and Supplementary Figures S1 to S11

## Data Availability

Anonymized data are available upon request from qualified academic investigators.

## Abbreviations

Aβ: Amyloid-beta
ACHE: Acetylcholinesterase
AD: Alzheimer’s disease
AGRN: Agrin
AMASS: Attention, Memory, and Aging Study at Stanford
APOE: Apolipoprotein E
ARSA: Arylsulfatase A
AUC: Area under Curve
CALB2: Calretinin
CCL3: CC chemokine ligand 3
CCL4: CC chemokine ligand 4
CCL11: CC chemokine ligand 11
CDR: Clinical dementia rating
CHI3L1: Chitinase-3 like-protein-1
CNS: Central nervous system
CRH: Corticotropin-releasing hormone
CSF: Cerebrospinal fluid
CSF2: Granulocyte-macrophage colony-stimulating factor
CST3: Cystatin C
CU: Clinically unimpaired
CV: Coefficient of variation
CXCL10: Chemokine ligand 10
CXCL8: Chemokine ligand 8
ENO2: Enolase 2
FABP3: Fatty acid-binding protein 3
FDR: False discovery rate
GDNF: Glial cell line-derived neurotrophic factor
GFAP: Glial fibrillary acidic protein
IC: Internal control
IFNG: Interferon gamma
IL5: Interleukin 5
IL6: Interleukin 6
IL8: Interleukin 8
IL9: Interleukin 9
IL10: Interleukin 10
IL16: Interleukin 16
IL33: Interleukin 33
IQR: Interquartile range
IPC: Inter-plate control
LOD: Limit of detection
MAPT: Microtubule-associated protein tau
MMSE: Mini-Mental State Examination
MRI: Magnetic resonance imaging
NEFH: Neurofilament heavy chain
NEFL: Neurofilament light chain
NPQ: NULISA protein quantification
NPTX1: Neuronal pentraxin-1
NPTX2: Neuronal pentraxin-2
NULISA: Nucleic acid-linked Immuno-Sandwich Assay
NULISAseq: NULISA with next-generation sequencing readout
PET: Positron emission tomography
PRDX6: Peroxiredoxin 6
p-tau: Phosphorylated tau
ROC: Receiver operating characteristic
ROI: Region of interest
S100A12: S100 calcium-binding protein A12
SD: Standard deviation
SAA1: Serum amyloid A1
SAMS: Stanford Aging and Memory Study
Simoa: Single-molecule array
SNAP-25: Synaptosomal-associated protein, 25kDa
SUVR: Standardized uptake value ratio
TDP-43: TAR DNA binding protein 43
TIMP3: Metalloproteinase inhibitor 3
TNF: Tumor necrosis factor
TREM1: Triggering receptor expressed on myeloid cells 1
TREM2: Triggering receptor expressed on myeloid cells 2
YWHAZ: 14-3–3 Protein zeta/delta
VSNL1: Visinin-like protein 1
