# Supplementary material for: Plasma proteomic signatures of preclinical Alzheimer’s disease in clinically unimpaired older adults
Source: Mol Neurodegener. 2026 Apr 24;21:31. doi: 10.1186/s13024-026-00941-5 (PMC13244826; doi:10.1186/s13024-026-00941-5)
Supplement: Supplementary file 1 — Supplementary Material 1: Additional file 1: Supplementary Tables S1-S4 and Supplementary Figures S1 to S11 [file 13024_2026_941_MOESM1_ESM.pdf]

Supplementary Table 1: Comparison between genetic *APOE* assignment and NULISAseq *APOE* assignment in SAMS

		NULISAseq *APOE* assignment	
		APOE4-	*APOE4+*
Genetic *APOE* Assignment	*APOE4-*	147	0
	APOE4+	0	46
		Concordance Rate	100.00%

Supplementary Table 2: Performance of NULISA and Lumipulse plasma biomarkers for discrimination of CSF A status in SAMS

Assay	AUC	Sensitivity	Specificity	PPV	NPV	Threshold	CI, lower	CI, upper
pTau217/Aβ42 (N)	0.940	0.857	0.968	0.909	0.948	-1.212	0.885	0.995
pTau217/Aβ42 (L)	0.907	0.829	0.894	0.744	0.933	0.006	0.849	0.966
Aβ42/Aβ40 (L)	0.893	0.943	0.819	0.660	0.975	0.093	0.825	0.961
pTau217 (N)	0.879	0.829	0.894	0.744	0.933	11.339	0.798	0.960
pTau217 (L)	0.838	0.800	0.840	0.651	0.919	0.171	0.755	0.922
Aβ42/Aβ40 (N)	0.779	0.943	0.596	0.465	0.966	0.409	0.702	0.857
pTau181 (N)	0.720	0.629	0.787	0.524	0.851	12.976	0.620	0.820
GFAP (L)	0.701	0.686	0.617	0.400	0.841	52.550	0.602	0.800
GFAP (N)	0.692	0.457	0.872	0.571	0.812	15.026	0.589	0.795
pTau181 (L)	0.649	0.914	0.330	0.337	0.912	1.305	0.543	0.756
NFL (L)	0.545	0.371	0.766	0.371	0.766	22.215	0.431	0.659
NFL (N)	0.536	0.457	0.660	0.333	0.765	14.252	0.422	0.651

Abbreviations: AUC = Area under the curve; CI = confidence interval; L= Lumipulse; N= NULISA; NPV= negative predictive value; PPV= positive predictive value

Supplementary Table 3: Performance of NULISA brain-derived and total plasma biomarkers for discrimination of PET A status in AMASS

Assay	AUC	Sensitivity	Specificity	PPV	NPV	Threshold	CI, lower	CI, upper
BD-pTau181	0.920	0.810	0.899	0.630	0.957	13.421	0.865	0.974
BD-pTau217	0.920	0.857	0.909	0.667	0.968	11.685	0.847	0.992
pTau217/Aβ42	0.865	0.762	0.848	0.516	0.944	-1.884	0.764	0.965
pTau217	0.861	0.714	0.909	0.625	0.938	11.574	0.773	0.948
BD-pTau231	0.855	0.810	0.869	0.567	0.956	13.152	0.762	0.948
pTau231	0.850	0.952	0.636	0.357	0.984	12.729	0.777	0.923
pTau181	0.763	0.762	0.717	0.364	0.934	12.721	0.659	0.867
Aβ42/Aβ40	0.735	0.905	0.535	0.292	0.964	2.015	0.630	0.840
GFAP	0.646	0.714	0.586	0.268	0.906	12.702	0.516	0.777
NFL	0.582	0.714	0.495	0.231	0.891	11.526	0.445	0.718

Abbreviations: AUC = Area under the curve; BD = brain-derived; CI = confidence interval; NPV= negative predictive value; PPV= positive predictive value.

Supplementary Table 4: Characteristics of the SAMS tau PET sample

	**SAMS CU (n = 71)**
**Age, mean (SD) y**	73.5 (6.96)
**Sex, n (%) female**	38 (53.5%)
**Education, mean (SD) y**	16.6 (2.14)
***APOE* genotype**	
ε2/ε3	6 (8.5%)
ε2/ε4	3 (4.2%)
ε3/ε3	50 (70.4%)
ε3/ε4	10 (14.1%)
ε4/ε4	2 (2.8%)
**Amyloid Status** (n Aβ+ / n total)	13 / 35 (37.1%)
**Race, n (%)**	
Asian	7 (9.9%)
Black or African American	1 (1.4%)
White	59 (83.1%)
More Than One Race	2 (2.8%)
Unknown/Not Reported	2 (2.8%)
**Ethnicity, n (%)**	
Hispanic or Latino/a	2 (2.8%)
NOT Hispanic or Latino/a	69 (97.2%)
**Plasma, PET lag, mean (SD) y**	0.415 (0.544)

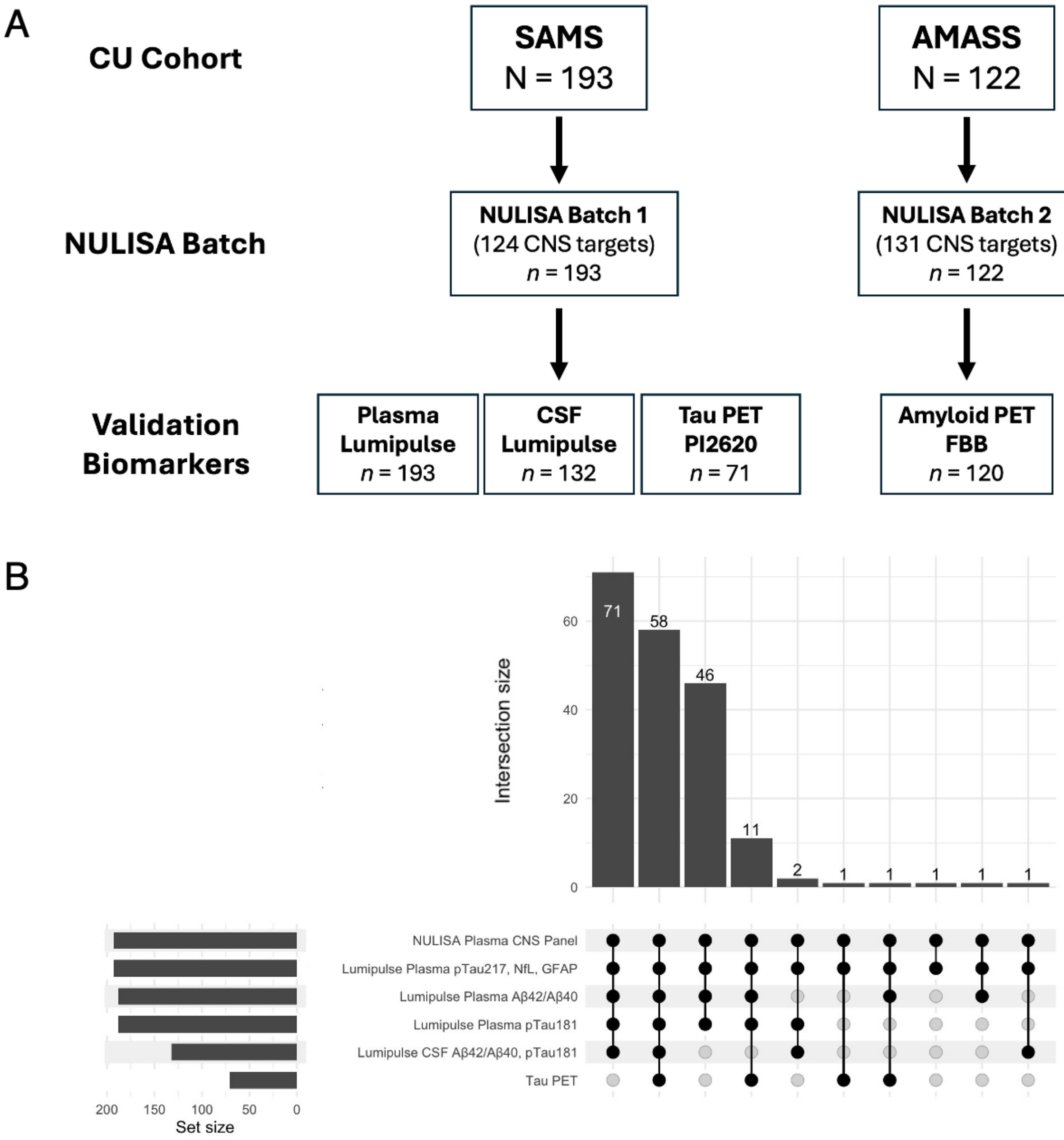

Supplementary Fig. 1 Overview of data availability by cohort. **A** Flow chart illustrating plasma data and biomarker validation data available in SAMS and AMASS. **B** Upset plot illustrating overlap across different biomarker modalities in SAMS, including between NULISA plasma, Lumipulse plasma pTau217, pTau181, and Aβ42/Aβ40, Lumipulse CSF Aβ42/Aβ40 and pTau181, and PI2620 Tau PET.

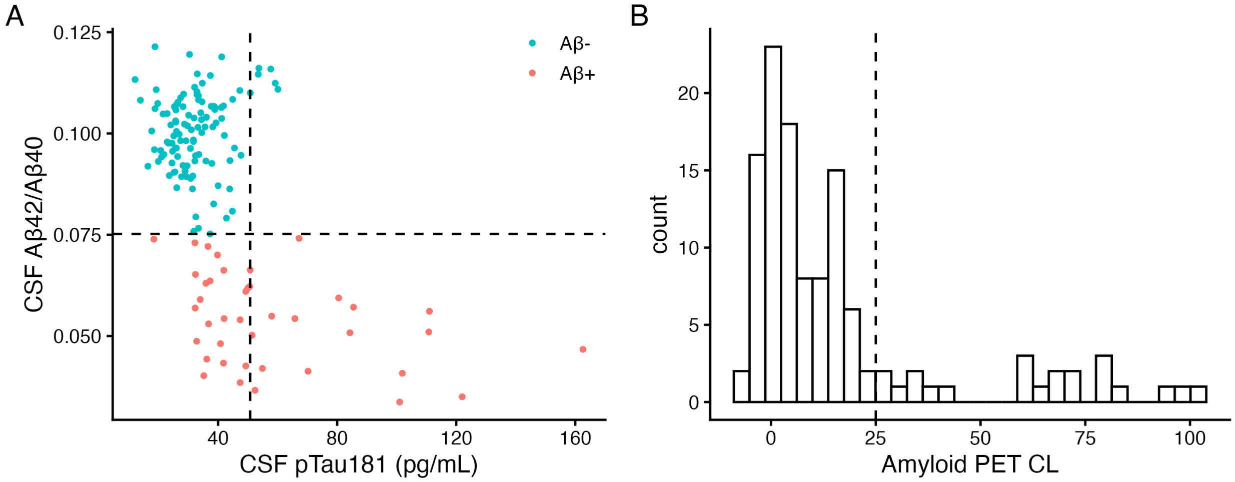

Supplementary Fig. 2 Validation biomarkers and positivity thresholds in SAMS and AMASS.
A Scatterplot illustrating distributions and cut-offs for CSF-defined A and T groups in the SAMS baseline CU sample (*n* = 153). Amyloid positivity was defined as < 0.0752 and Tau positivity was defined as pTau181 > 50.77, corresponding to 2SD above the mean of the A- group.
B Amyloid PET distribution in AMASS CU (n=120). Amyloid PET positivity was defined as CL > 25.

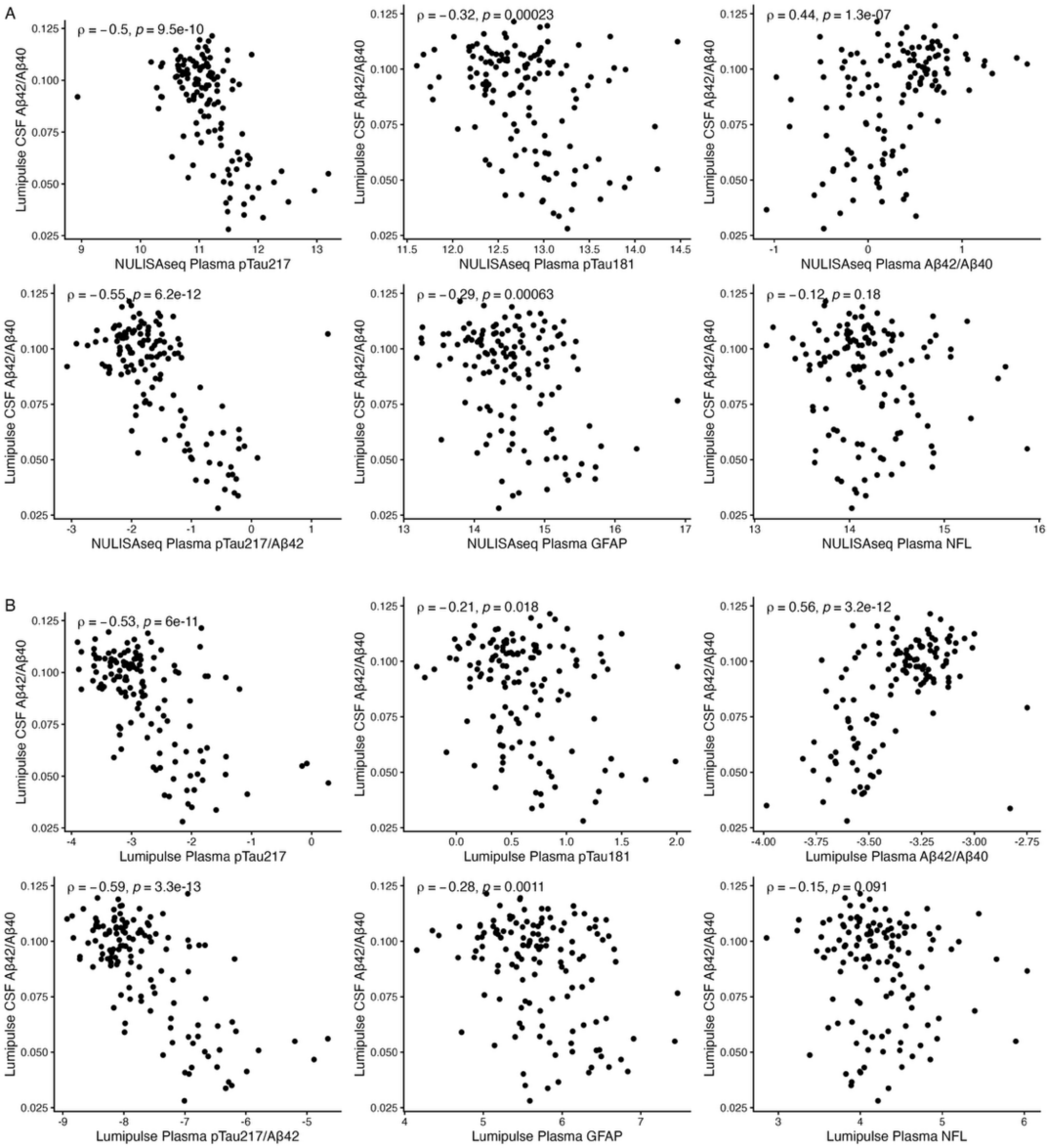

Supplementary Fig. 3 Associations between CSF Aβ42/Aβ40 and core AD-relevant proteins in SAMS **A** Scatterplots illustrate correlations between CSF Aβ42/Aβ40 and plasma proteins measured using NULISA (*n*=132) and **B** Lumipulse (pTau217, GFAP, NfL *n*=132; pTau181 *n*=131; Aβ42/Aβ40 *n*=129) immunoassays. Spearman (*rho*) correlation coefficients and *p*-values are shown. NULISAseq proteins are in NPQ units and Lumipulse proteins measured in pg/mL were log2 transformed for comparison to NULISAseq measurements.

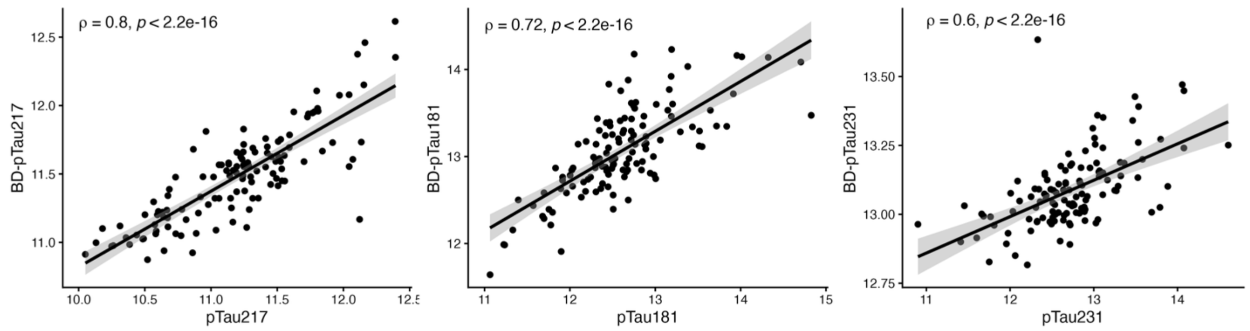

Supplementary Fig. 4 Correlation between brain-derived and total NULISAseq plasma pTau isoforms. Scatterplots illustrate the distributions and correlations between brain-derived and total pTau assays in the AMASS cohort (*n*=122). NULISAseq proteins are in NPQ units. Spearman (ρ) correlation coefficients and *p*-values are shown along with the best fit line and 95% CI. BD: brain-derived; NPQ: NULISA protein quantification unit.

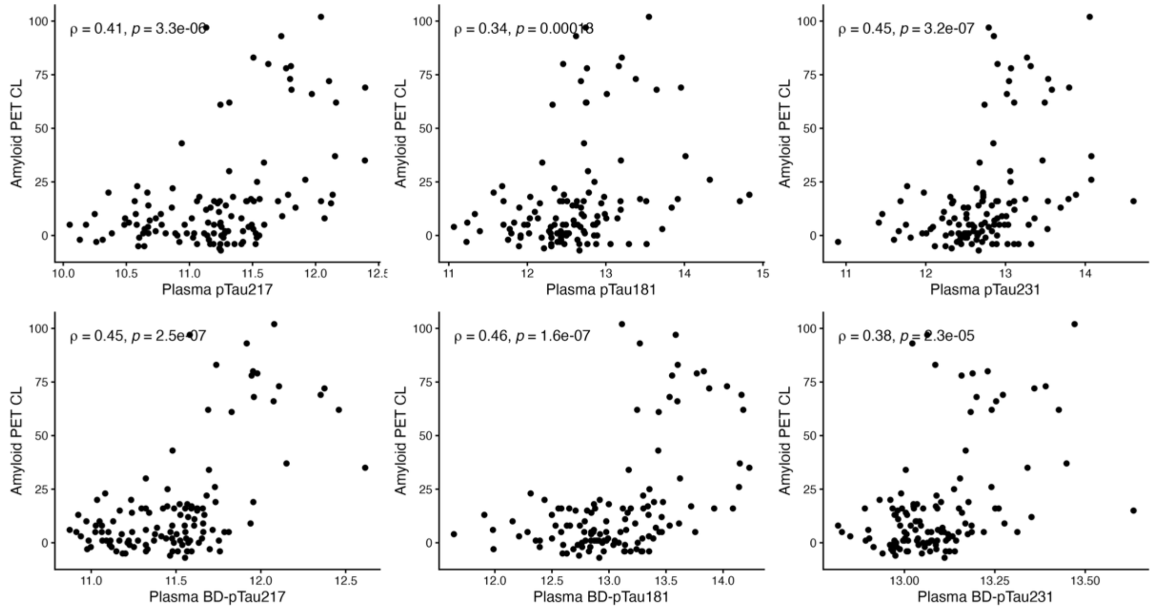

Supplementary Fig. 5 Association between brain-derived and total NULISAseq plasma pTau assays and amyloid PET. Scatterplots illustrate the distributions and correlations between amyloid PET CL and brain-derived and total pTau concentrations in the AMASS cohort (*n*=120). NULISAseq proteins are in NPQ units and amyloid PET is in centiloids (CL). Spearman (ρ) correlation coefficients and *p*-values are shown. BD: brain-derived; NPQ: NULISA protein quantification unit.

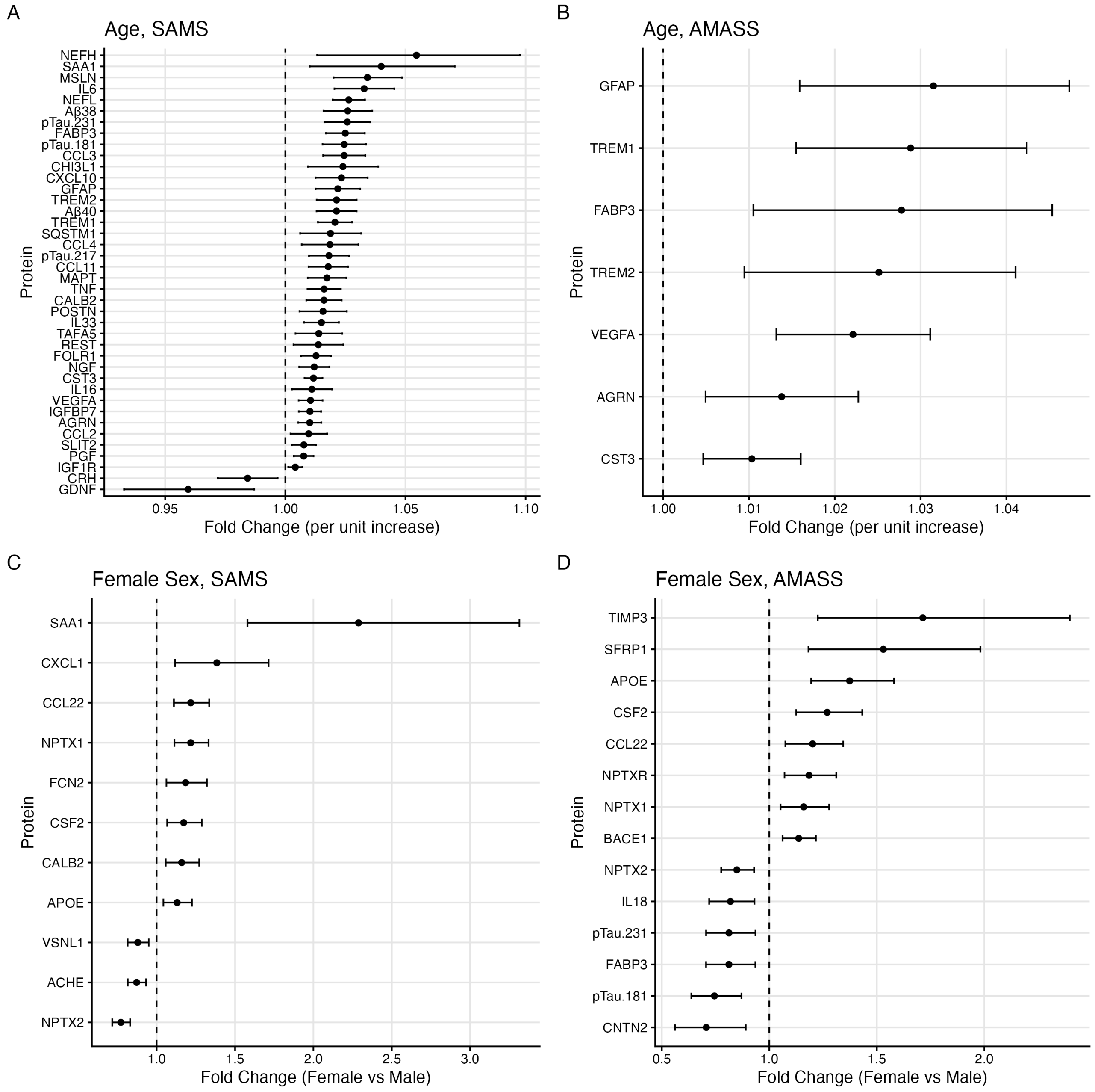

Supplementary Fig. 6 NULISA CNS targets associated with age and sex. **A-D** Forest plots illustrate fold change differences (mean and 95% CI) for NULISA targets that were differentially expressed (FDR-corrected $p < 0.05$) as a function of age and sex in SAMS (**AC**, n=193) and AMASS (**BD**, n=122).

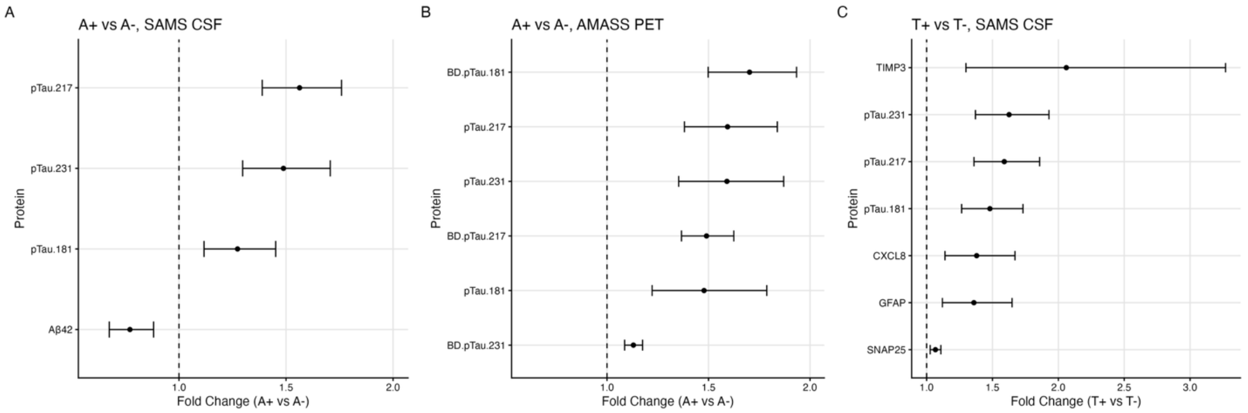

Supplementary Fig. 7 NULISA CNS targets associated with A and T biomarkers. **A-C** Forest plots illustrate fold change differences (mean and 95% CI) for significant NULISAseq targets (FDR-corrected $p < 0.05$, controlling for age and sex) as a function of CSF-defined A and T status in SAMS (**AC**, n=132) and PET-defined A status in AMASS (**B**, n=120).

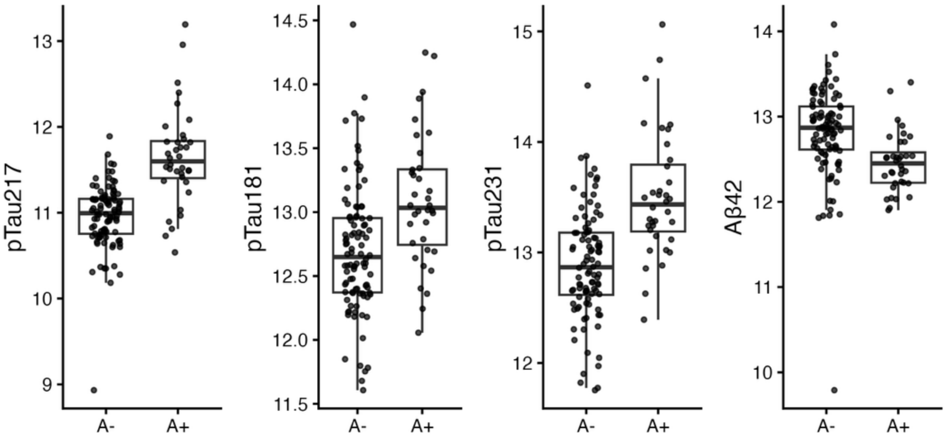

Supplementary Fig. 8 NULISA CNS targets associated with A status in SAMS. Boxplots illustrate NPQ distributions for significant targets (FDR-corrected $p < 0.05$, controlling for age and sex) by CSF-defined A status in SAMS (A-, n=96; A+, n=36).

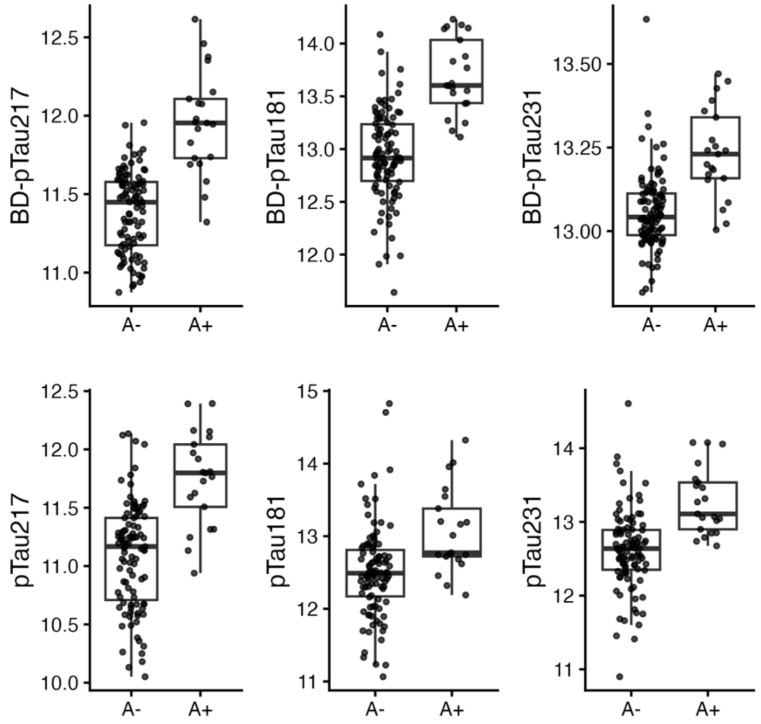

Supplementary Fig. 9 NULISA CNS targets associated with A status in AMASS. Boxplots illustrate NPQ distributions for significant targets (FDR-corrected $p < 0.05$, controlling for age and sex) by PET-defined A status in AMASS (A-, n=98; A+, n=22).

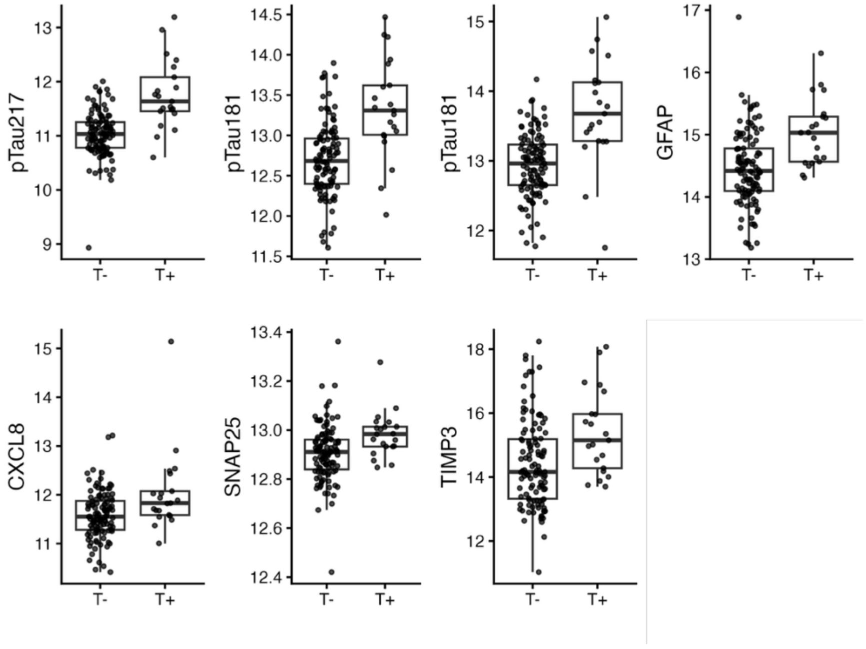

Supplementary Fig. 10 NULISAseq targets associated with T status in SAMS. Boxplots illustrate NPQ distributions for significant CNS targets (FDR-corrected $p < 0.05$, controlling for age and sex) by CSF-defined T status in SAMS (T-, n=111; T+, n=21).

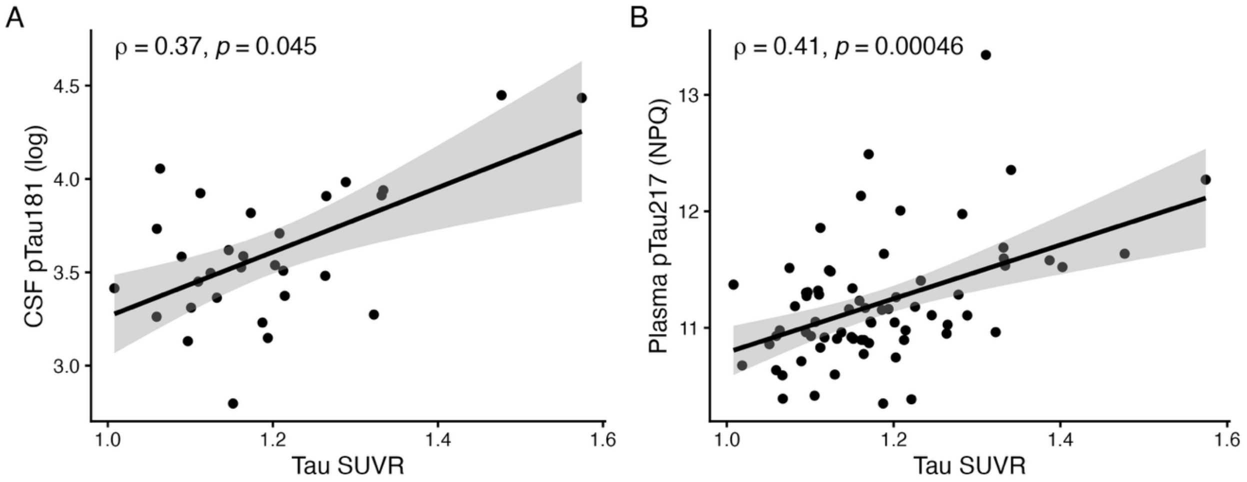

Supplementary Fig. 11 Association between temporal cortex tau accumulation and fluid biomarkers. **A** Scatterplots illustrate the distributions and correlations between temporal cortex tau SUVR and CSF pTau181 (log transformed) and **B** NULISAseq plasma pTau217 (NPQ). Spearman (ρ) correlation coefficients and *p*-values are shown, together with the best fit line and 95% confidence band.
